# Self-Organized Attractor Dynamics in the Developing Head Direction Circuit

**DOI:** 10.1016/j.cub.2018.01.010

**Published:** 2018-02-19

**Authors:** Joshua P. Bassett, Thomas J. Wills, Francesca Cacucci

**Affiliations:** 1 Department of Neuroscience, Physiology and Pharmacology, University College London, Gower Street, London WC1E 6BT, UK; 2 Department of Cell and Developmental Biology, University College London, Gower Street, London WC1E 6BT, UK

**Keywords:** head direction cells, development, anterodorsal thalamic nucleus, attractor network

## Abstract

Head direction (HD) cells are neurons found in an extended cortical and subcortical network that signal the orientation of an animal’s head relative to its environment [1, 2, 3]. They are a fundamental component of the wider circuit of spatially responsive hippocampal formation neurons that make up the neural cognitive map of space [4]. During post-natal development, HD cells are the first among spatially modulated neurons in the hippocampal circuit to exhibit mature firing properties [5, 6], but before eye opening, HD cell responses in rat pups have low directional information and are directionally unstable [7, 8]. Using Bayesian decoding of HD cell ensemble activity recorded in the anterodorsal thalamic nucleus (ADN), we characterize this instability and identify its source: under-signaling of angular head velocity, which incompletely shifts the directional signal in proportion to head turns. We find evidence that geometric cues (the corners of a square environment) can be used to mitigate this under-signaling and, thereby, stabilize the directional signal even before eye opening. Crucially, even when directional firing cannot be stabilized, ensembles of unstable HD cells show short-timescale (1–10 s) temporal and spatial couplings consistent with an adult-like HD network. The HD network is widely modeled as a continuous attractor whose output is one coherent activity peak, updated during movement by angular head velocity signals and anchored by landmark cues [9, 10, 11]. Our findings present strong evidence for this model, and they demonstrate that the required network circuitry is in place and functional early during development, independent of reference to landmark information.

## Highlights

• Non-visual cues can anchor head direction (HD) cells in pre-eye-opening rat pups
• Internal network dynamics are preserved even when the HD representation is unstable
• Angular velocity under-signaling drives instability, which is mitigated by corners
• Circuit architecture develops even before any landmarks can stabilize HD responses

## Teaser

Bassett et al. show that the attractor architecture of the head direction (HD) circuit develops without reference to landmarks. Non-visual cues can stabilize HD responses in rat pups aged 13 days or older. Instability in pre-visual HD cells is caused by angular velocity under-signaling, mitigated by proximity to corners in square enclosures.

## Results and Discussion

### Local Cues Can Stabilize Developing HD Networks from Post-natal Day 13 Onward

**Figure 1:**
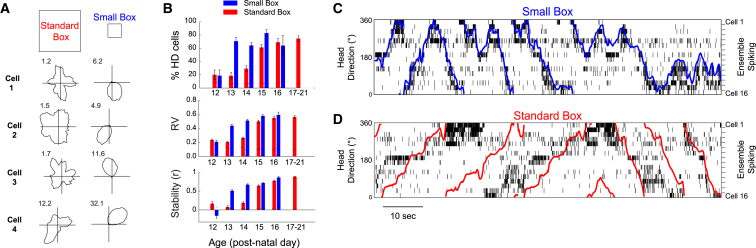
HD Cell Signaling Is Stabilized When Rats Explore a Small (20 cm Side) Environment, from P13 Onward (A) Example firing rate polar plots for 4 HD cells recorded at P13 in the standard (left) and small (right) boxes. Numbers at top left indicate peak firing rate (Hz). (B) HD cells are more numerous (top) and have a higher spatial tuning (Rayleigh vector [RV], middle) and intra-trial stability (bottom) when recorded in a small versus a standard box in animals older than P13. Error bars indicate SEM. See also Figure S1. (C and D) HD cell network internal organization is preserved in the standard box, even when directional signaling is unstable. Colored traces indicate the rat’s actual head direction, overlaid upon spike raster plots for all simultaneously recorded HD cells, in the small (C) and standard (D) boxes. For both (C) and (D), HD cells are ordered vertically by their preferred firing direction in the small box. The sequences of HD cell activation for each head turn direction are similar in the small and standard boxes, but the direction signaled by HD cell firing consistently undershoots actual rotation in the standard box.

Previous studies recorded head direction (HD) cells from rat pups moving within a 62 × 62-cm open-field environment (standard box) typical of that used with adult animals [5, 7]. Here we also introduced animals to a second smaller environment (small box, 20 × 20 cm). We recorded anterodorsal thalamic nucleus (ADN) neurons (1,276 cells from 21 animals, aged post-natal day (P)12–P21; see Figure S1A for anatomical location of recording sites) while rat pups alternately explored the two environments (Figure 1A). The proportion, spatial tuning, and stability of HD cells were higher in the small versus the standard box (Figures 1B and S1C), most markedly at the pre-visual ages P13–P14 (2-sample Z-test for proportions, Z > 7.5, p < 0.0001 for both P13–P14; ANOVA Rayleigh vector [RV], Age^∗^Env F_4,1025_ = 15, p < 0.001, simple main effects (SME)_(ENV)_ p < 0.001 at P13–P14; ANOVA stability, Age^∗^Env F_4,1019 =_ 45, p < 0.001, SME_(ENV)_ p < 0.001 at P13–P14). There was no difference between the proportions of neurons classified as HD cells in the small box at P13 and those at P17–P21, when the HD system was fully mature (Z = 1.1, p = 0.27). Sensory modalities other than vision are, therefore, capable of anchoring HD representations before eye opening. Angular head velocity did not differ significantly between environments, excluding this as cause for increased stability in the small box (Figure S1B).

At P13–P14, the internal organization of the HD network appeared to be preserved even when HD responses were unanchored to the environmental reference frame (standard box): for each head turn direction, similar spiking sequences could be observed across co-recorded HD cells in both small and standard boxes. Network activity transitioned smoothly through directional space, while often undershooting actual head movements (Figure 1C).

### HD Cells in Developing Rats Display Adult-like Spatial and Temporal Coupling, Even When Drifting

**Figure 2:**
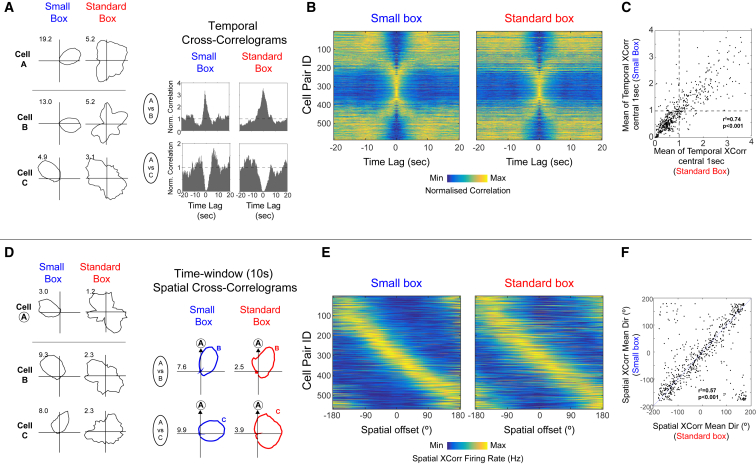
Short-Timescale Temporal and Spatial Couplings between HD Cells Are Preserved Even When Directional Signaling Is Unstable (A) Example polar plots (left) and temporal cross-correlograms (TXCs, right) for 3 HD cells recorded in small and standard boxes. (B) TXCs of all co-recorded HD cell pairs in the small (left) and standard (right) boxes, normalized between their minimum (dark blue) and maximum (yellow) values. Each row in the image shows one TXC, and rows are sorted on the basis of preferred firing direction difference in the small box. (C) Correlation between mean values of the central 1 s of TXCs in the small versus standard box. (D) Example polar plots (left) and time-windowed (10 s) spatial cross-correlograms (SXCs, right) for 3 HD cells. (E) SXCs of all co-recorded HD cell pairs in the small (left) and standard (right) boxes. HD pairs are sorted as in (D). (F) Circular-circular correlation between the mean directions of SXCs in the small versus standard box.

To confirm whether the internal organization of the HD network is preserved, even when its responses are unanchored from the external environment, we examined the short-term temporal and spatial couplings between pairs of co-recorded neurons. We computed temporal cross-correlograms (Figures 2A and 2B) and time-windowed spatial cross-correlograms (Figures 2D and 2E) for all pairs of co-recorded cells that displayed HD tuning in the small box (see the STAR Methods). To eliminate residual HD stability in the standard box as a confounding factor, we excluded any cells that displayed significant HD tuning in the standard box. Despite this, both the temporal and spatial relationships between pairs of co-recorded HD cells were preserved across both the small and standard environments (significantly correlated temporal and spatial offsets between small and standard boxes, see Figures 2C and 2F). This demonstrates that, even when HD cells are unstable in the open field (on P13–P14), the internal network structure is unchanged.

### Attractor Connectivity Precedes HD Landmark Stabilization

**Figure 3:**
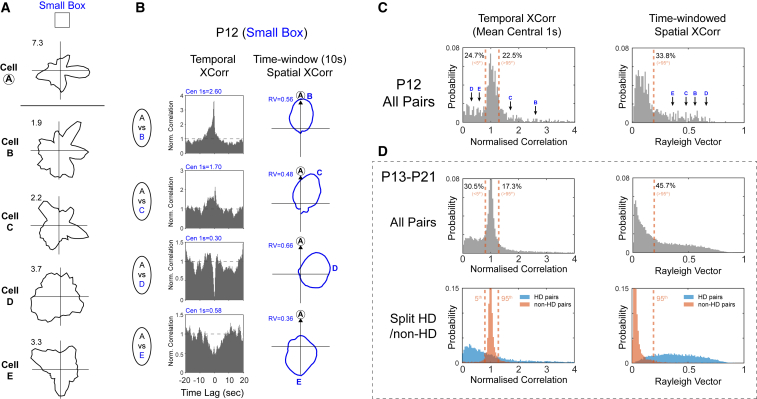
HD Attractor Network Organization Is Present at P12, Even before HD Cells Can Be Anchored to Landmarks (A) Polar plots for five example cells recorded in a small box at P12, showing no HD tuning over a 10-min session. (B) Temporal cross-correlograms (TXCs; left) and spatial cross-correlograms (SXCs; right) between cell A and cells B–E. Blue text at top left shows mean of central 1 s of TXC and RV length of SXC, respectively, for each example. (C) Probability distributions of mean of central 1 s of the TXC (left) and RV length of the SXC (right) scores for all P12 co-recorded cell pairs (n = 452). Black arrows with blue letters indicate scores from examples shown in (A) and (B). Orange dashed lines show the values of the fifth and 95^th^ percentiles of scores of known non-HD cells in older animals (see D; only 95^th^ percentile is shown for SXC). Black text refers to percentages of P12 scores above or below these percentiles. (D) Top row: As for (C) but for all co-recorded cell pairs P13–P21. Bottom row: Same data as top row but distributions of HD-HD pairs (light blue) and nonHD-nonHD pairs (orange) are plotted separately. Orange dashed lines shown here and in (C) and (D) (top row) are derived from the fifth and 95^th^ percentiles of the orange (nonHD-nonHD) distributions. See also Figure S2.

Introducing the youngest group of animals (P12) to the small box did not result in an improvement in HD cell stability/tuning (Z-test for %HD, Z = 0.71, p = 0.45; SME_(ENV)_ for RV, p = 0.99; for stability, small box lower; Figure 1B). Nevertheless, many co-recorded cell pairs showed temporal and spatial coupling indicative of an attractor neural structure, even at this age (Figures 3A–3C). To test whether the degree of temporal and/or spatial coupling at P12 was significantly higher than chance, we measured the proportions of P12 cross-correlogram scores lying beyond 95% confidence limits for HD-HD coupling (defined as the fifth and 95^th^ percentiles of the scores from all known non-HD cell pairs in older rats; Figure 3D). The proportion of P12 cell pairs with coupling scores beyond these confidence limits significantly exceeded 5% (one sample Z-test; temporal, Z = 19, p < 0.001 [fifth], Z = 14, p < 0.001 [95^th^]; spatial, Z = 33, p < 0.001), demonstrating that HD neurons display fixed temporal and spatial offsets at P12, an age at which none of the tested experimental manipulations resulted in environmentally stable HD responses. The presence of these temporal and spatial couplings cannot be explained by the overall broader tuning of HD cells at younger ages (Figure S2).The HD network thus displays a key signature of continuous attractor structure before HD responses can be stabilized by local landmarks.

### Drift of the HD Representations Occurs due to Systematic Under-Signaling of Angular Head Velocity

**Figure 4:**
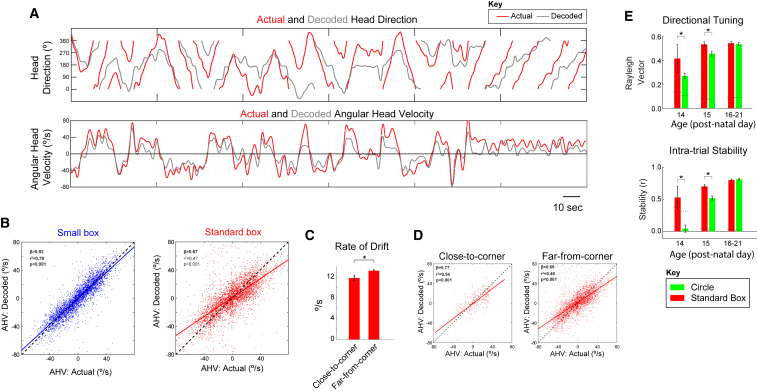
HD Drift in Young Rodents Is Caused by Angular Head Velocity Under-Signaling and Is Reduced by Proximity to Corners (A) Example of actual (red) and decoded (gray) head direction (top) and angular head velocity (bottom) values displayed by a P14 rat during 5 min exploration in the standard box. See also Figure S3. (B) Correlation between actual (x axis) and decoded (y axis) angular head velocity scores in the small (left) and standard (right) boxes across all decoded ensembles (n = 6). Slope of relationship between actual and decoded angular head velocity is significantly smaller in the standard versus small box. (C) Mean (±SEM) rate of drift (rate of divergence between actual and decoded head direction) when rats were close or far from the corners of the standard box. (D) Angular head velocity under-signaling is reduced when rats are close to corners. Correlations are shown between actual and decoded angular head velocity scores in the standard box, split by proximity to corners (left, close; right, far). See also Figure S4. (E) Directional tuning and intra-trial stability of HD cells are reduced in a circular environment, as compared to the standard (square) box, on P14–P15. Bar charts show the mean (±SEM) Rayleigh vector (top) or intra-trial stability (bottom) of HD cells recorded in standard and circular environments.

To further characterize the temporal dynamics of drifting HD networks, we applied a cross-trial Bayesian decoding approach, using firing rate maps of stable HD cells in the small box to decode signaled direction (in small box coordinates, see the STAR Methods) as the rat moved in the standard box, at P13–P14. Although, as expected, decoded headings diverged from actual headings, decoding produced a coherent estimate of direction exhibiting continuous, smooth transitions during a trial, consistent with attractor dynamics (Figure 4A, top; Figure S3). The coherence and smoothness of decoded trajectories were not significantly different whether decoding was performed on standard or small box data, further demonstrating the maturity of the internal network dynamics, despite ongoing drift (Figures S3E and S3F). We then obtained the decoded angular head velocity by calculating the first derivative of the decoded head direction (angular head velocity, Figure 4A, bottom; see the STAR Methods). We found that, when HD responses are drifting, although angular head velocity is linearly correlated with actual head velocity, it is under-signaled by drifting HD networks in the standard box (Figure 4B, regression β significantly lower for standard box, t_(6916)_ = 16.54, p < 0.0001). Thus, the most prominent source of error in young HD networks is a systematic under-signaling of angular head velocity, which cannot be compensated for by alternative sensory cues in the standard box.

### Geometric Cues Can Mitigate Path Integration Error

Introducing rat pups in the small box results in stabilization of HD responses (Figures 1A, 1B, and S1C) and reduction of angular head velocity under-signaling (Figure 4B, left). Following previous findings that boundaries stabilize place fields during post-natal development [12] and grid cell signaling in adult mice [13], we tested whether HD cells were more stable when animals were close to an environmental boundary. Unexpectedly, the rate of drift (rate of divergence between actual and decoded heading) was significantly greater close to walls (mean rate of drift: walls = 13.3 ± 0.52 degrees/s, center = 12.2 ± 0.70 degrees/s; t_(3476)_ = 2.5, p = 0.007). This is likely caused by an increase in actual angular head velocity when animals are close to walls (mean angular head velocity: walls = 21.6 ± 0.6 degrees/s, center = 20.2 ± 0.8 degrees/s; t_(3476)_ = 2.70, p = 0.012). However, further analyses showed that both the rate of drift and angular head velocity under-signaling were significantly reduced when animals were close to a corner (mean rate of drift: t_(3476)_ = 1.98, p = 0.048; see Figures 4C and S4G; angular head velocity under-signaling difference of β: t_(3476)_ = 2.47, p = 0.013; see Figure 4D). This effect cannot be accounted for by differences in the number of samples or differences in actual angular head velocity close to and far from corners (Figures S4A–S4D). Additional evidence suggests that angular head velocity under-signaling is further reduced when rats are close to corners and either face the corner or have their head in close proximity to the wall (Figures S4E and S4F). Corners may, therefore, be a geometric feature used to offset path integration error in developing animals.

To further investigate the stabilizing influence of corners on the developing HD network, we introduced rat pups (P14–P15) to a circular environment of similar dimensions to the standard square environment (matched perimeter). When recorded in the circular environment, HD cells showed reduced directional tuning and intra-trial stability (Figures 4E and S4H; directional tuning [RV]: ANOVA Age^∗^Env F_(2,348)_ = 8.5, p < 0.001; SME_(ENV)_: P14, p = 0.023; P15, p < 0.001; stability: ANOVA Age^∗^Env F_(2,343)_ = 18.9, p < 0.001; SME_(ENV)_: P14, p < 0.001; P15, p < 0.001). These findings further support the interpretation that corners may be used to stabilize HD cells in early development.

A striking characteristic of HD cells is that, although their *absolute* preferred firing directions can change (following disorientation or manipulations of the environment), the tunings of simultaneously recorded cells retain their *relative* spatial offsets under all circumstances [14, 15]. This experimental finding underlies all available models of the HD circuit, describing it as possessing 1-dimensional ring architecture, with the connectivity between HD units arranged such that nodes with similar heading preferences are mutually excited, whereas nodes with divergent preferred directions inhibit each other [9, 10, 11]. This topology gives rise to continuous attractor dynamics, whereby the network always produces a single, localized peak of activity involving neighboring nodes, which represents the current directional bearing. In adult mice, HD cell firing during sleep is consistent with attractor dynamics [16], and HD correlates are preserved during spindles in rats, without movement or landmark input [17]. The position of the activity peak is thought to be updated in response to head movement by the activity of upstream angular velocity-responsive cells, which have been described in the rat midbrain [18, 19]. In this model, the network integrates directional position from velocity, to support the angular component of path integration behaviors. Indeed, lesions of the HD system significantly impair performance on spatial tasks that depend on angular path integration [20, 21].

Path integration is prone to overwhelming accumulation of error without periodic reference to stable external cues, which discharge the error and anchor the spatial representation to the environment [9, 10, 11]; if the waking HD system is both an attractor and a path integrator, its development requires not only the intrinsic ring topology but also a self-motion input (e.g., angular head velocity) and landmark cues corresponding to stable features of the environment. Theoretical models of the emergence of the HD system suggest the presence of a distal landmark cue that acts as a supervisory input, simultaneously anchoring the network to the external world and instructing the correct wiring across nodes [22, 23]. Previous studies of HD network development have shown that, even before eye opening, when HD cells exhibit stable tuning within a trial, they also retain their relative spatial offsets between trials, consistent with the behavior of adult HD cells [8]. However, this observation alone cannot address the central question of whether the internal organization of the circuit precedes the emergence of stable directional signaling or rather, as predicted by theoretical models of HD circuit development, these two features of the network arise concomitantly [22, 23].

Here we show that, during early post-natal development (P12–P14) before eye opening, the internal dynamics of the HD circuit, when observed at fine timescales, are exactly as described in a mature continuous attractor network, even while the circuit’s spatial relationship to the environment is completely adrift from moment to moment. The internal organization of the HD circuit therefore appears to be in place before its ability to represent heading direction is manifested. Our results provide new constraints on models of continuous attractor network development, as they demonstrate that the rigid coupling of spatial relationships across directional nodes is not extracted from the structure of sensory input through a learning process [22, 23], but instead likely arises through internal, self-organized processes during development (with possible further refinement through learning [24]). This finding may also generalize to other putative attractor networks such as grid cells [25, 26, 27, 28], which display strong coupling as soon as they can be detected in young animals [29], and the recently discovered neural representation of direction in *Drosophila* [30, 31, 32, 33].

HD cells in the ADN reflect the influence of both ascending self-movement information from the tegmento-mammillary circuit and descending visual landmark information from cortical projections [34]. The reciprocal connections between the tegmental and mammillary nuclei as well as the mammillo-thalamic projections are developmentally complete by approximately P10 [35, 36]. Therefore, the generative tegmento-mammillary circuit in which the attractor architecture is thought to be expressed is structurally complete by the time we find attractor dynamics in the ADN, at P12, days before eye opening. Our present findings therefore suggest that the tegmento-mammillary circuit is both necessary for driving HD cell activity in the ADN, in line with the effects of lesions in the adult brain [37], and sufficient without a requirement for supervisory visual input even during its initial organization.

The HD attractor in young animals shows a linear response to angular head velocity but with reduced gain, indicating systematic under-signaling. This constitutes direct experimental evidence that the attractor’s activity peak is shifted around the ring proportionally to the magnitude of angular head velocity input [38], thereby integrating velocity to position as predicted by current models of the HD network. In the open field, the under-signaling of velocity, without the ability to correct the accumulating error with landmarks amounts to an open-loop state, resulting in drift of directional firing with respect to the environment. It remains undetermined whether the ultimate cause for this under-signaling can be imputed to reduced vestibular input [39], to its incomplete integration by the HD network, or a combination of the two.

Our results also suggest that geometrical features (corners) can be used to stabilize HD responses during early post-natal development, at a time when access to distal cues is still limited. A possible mechanism for this geometric feature detection, which could additionally explain the time course of its development between P12 and P13, is the progressive refinement of whisking at these ages [40]. An important question for future research is whether geometric input to HD cells is affected by environmental rearing conditions [41]. These results also provide new mechanistic insights as to the recognized importance of geometric features in the development of spatial cognition in children [42, 43].

## STAR★Methods

### Key Resources Table

**Table:** 

REAGENT or RESOURCE	SOURCE	IDENTIFIER
**Chemicals, Peptides, and Recombinant Proteins**		

Cresyl violet	Sigma Aldrich	C5042, http://www.sigmaaldrich.com/catalog/product/sigma/c5042?lang=en&region=US
Histoclear	National Diagnostics	HS-202, https://www.nationaldiagnostics.com/histology/product/histo-clear-ii
Thionin	Sigma Aldrich	88930, https://www.sigmaaldrich.com/catalog/product/sigma/88930

**Experimental Models: Organisms/Strains**		

Lister hooded rats	In house breeding (Charles River original source)	http://www.criver.com/products-services/basic-research/find-a-model/lister-hooded?loc=GB

**Software and Algorithms**		

Custom MATLAB routines	This paper	N/A
MATLAB	Mathworks. MA	RRID: SCR_001622, https://uk.mathworks.com/products/matlab.html

**Other**		

Single-screw microdrive	Custom made	N/A
Microwire (17um, platinum iridium)	California Fine Wire Company	Product code:100167, http://www.calfinewire.com/datasheets/100167-platinum10iridium.html
NanoZ plating equipment	Multichannel Systems	nanoZ, http://www.multichannelsystems.com/products/nanoz
Recording system (pre-amp and systems unit)	Axona	Product code: Dacq/USB64, http://axona.com/products
Omnetic connectors (microdrive assembly)	Genalog	Product code: A79026-001,http://genalog.com/genalog-linecard/omnetics/
2x16channel headstage preamplifiers	Axona	Product code: HS-116M1D, http://axona.com/products

### Contact for Reagent and Resource Sharing

Further information and requests for resources should be directed to and will be fulfilled by the Lead Contact, Francesca Cacucci (f.cacucci@ucl.ac.uk).

### Experimental Model and Subject Details

Subjects were 21 male Lister Hooded rats (RGD 2312466) aged P12-P21 weighing 18-29 g at the time of surgery. All procedures were approved by the UK Home Office, subject to the restrictions and provisions contained in the Animals Scientific Procedures Act of 1986. Litters were bred on site and implanted subjects remained with their mothers and litter-mates throughout the experimental period. Litters were housed in 42x32x21 cm cages furnished with nesting material and environmental enrichment objects, and maintained on a 12:12 hour light:dark schedule with lights off at 13:00. Litters were culled to 8 pups at P4 in order to minimize inter-litter variability. Implanted pups were separated from the litter for between 20 to 120 minutes per day for electrophysiological recordings. All procedures were conducted in accordance with the UK Animals Scientific Procedures Act (1986).

### Method Details

#### Surgery and electrodes

Rats were anaesthetised using 1%–3% isoflurane and buprenorphine via subcutaneous injection at 0.15mg/kg of body weight. Rats were implanted with 8 tetrodes consisting of HM-L coated 90% platinum/10% iridium 17 μm wire (California Fine Wire, Grover City, CA). The implanted apparatus weighed 1 g. Tetrode bundles were implanted into the ADN using the following stereotaxic coordinates: 1.7 mm posterior to bregma, 1.2mm lateral from the midline at bregma, and 4.2mm ventral from the skull surface at bregma. Following surgery, rats were placed on a heating pad until they could move spontaneously and then were returned to the home cage. After experiments were completed, tetrode position was confirmed by transcardially perfusing the rat (4% formaldehyde in PBS) while the tetrodes remained in their final position, followed by brain sectioning at 30μm, and Nissl-staining of the resulting sections. See Figure S1A for example sections.

#### Single-unit recording

Following surgery, rats were allowed 24 hours recovery. Tetrode bundles were then advanced ventrally in increments of 62.5-250 μm/day. Experimental recording sessions began when any single unit neural activity could be identified. Single unit data was acquired using the DACQ system (Axona Ltd, St. Albans, UK). Position and directional heading was recorded using a 2-point tracking system consisting of 2 LEDs spaced 7 cm apart and attached to the headstage amplifier in a fixed orientation relative to the animals’ head. Isolation of single units from tetrode-recorded data was performed manually on the basis of peak-to-trough amplitude or principal components, using the TINT software package (Axona Ltd., St Albans, UK) with the aid of KlustaKwik1 automated clustering [44].

#### Behavioral Testing

Single-unit recording trials took place in one of two square recording arenas. The ‘standard box’ had a side length of 62.5cm and was 50cm high, painted light gray, and placed on a black platform. The box was placed in the open laboratory, and distal visual cues were available in the form of the fittings and contents of the laboratory. The floor of the arena was not cleaned. There were no further polarizing cues placed within the recording arena. In order to ascertain whether cells which did not display a stable HD correlate in the standard box could be anchored to the laboratory frame in any other environment, we recorded the activity of the cells in a smaller square box (‘small box’: 20cm side length, 42cm high). The small box was placed on the same platform as the standard box and centered in the same location. In the majority of small box trials, the small box contained two polarizing cues, a 3D piece of wood (2cm x 4cm x 42cm), placed in the NW corner, and a sheet of polystyrene covering the E wall. Rats were subject to between 1 and 4 standard box trials (median 2), and 1 to 5 small box recording trials (median 2) per session (maximum total trials 7, median 3). As HD cells in the standard box were already mature by P15-16 (see also [5, 6, 7]), the oldest rats (P17-21) were not tested in the small box. Rat pups were kept in a separate holding box (40 × 40 × 25cm) furnished with bedding and a heating pad in between recording trials. A sub-set of rats (N = 14, contributing 351 HD cells) were also tested in a circular environment, from P14 onward. The circular environment was 79cm diameter, and (similarly to the standard box) was wooden, painted light gray, and placed on a black platform. A large plain white cue card (75cm x 1 m), illuminated by a 40W lamp was placed outside the environment, approximately 75cm away from its edge. No polarizing cues were placed inside the environment.

### Quantification and Statistical Analysis

#### Construction of firing rate maps

To minimize artifactual correlates due to under-sampling of position, data was only included if the angular path length for the session exceeded the equivalent of 25 full head turns (values derived from the 5^th^ percentile of the whole dataset). Positional (directional) data was sorted into 6° bins in the yaw plane. Following this, total dwell time, d, and spike count, s, for the whole trial was calculated for each directional bin. The binned position dwell time and spike count maps were then smoothed using a 30° boxcar filter, and the rate for each directional bin is defined as s/d.

#### Classification of single-units as HD cells

To minimize artifactual correlates due to under-sampling, only cells which fired at least 100 spikes in a recording session were included in further analysis. Single units were classified as HD cells if the mean resultant vector length (Rayleigh vector; RV) of the directional firing rate map exceeded a threshold defined as the 95th percentile of a population of RV scores derived from age- and brain area-matched spatially shuffled data [5]. Briefly, shuffled data was generated by shifting spike trains relative to position by a random amount between 20 s and trial duration minus 20 s, leaving the temporal structure of the spike train and the positional data otherwise unchanged. The shuffled data was then used to construct directional rate maps, as described above. This process was repeated a sufficient number of times for there to be 100,000 shuffled RV values for every 1-day age group, for each brain area. Single units with an RV ≥ 95th percentile of this shuffled population were defined as HD cells.

#### Quantitative analysis of directional signaling

‘*% HD cells’* was defined as the number of neurons classified as HD cells divided by number of total cells recorded in an environment (standard or small box). The difference between proportions of HD cells in the standard and small boxes in each age bin was tested using a 2-sample Z-test for proportions. ‘*Intra-trial stability’* was defined as the correlation between spatially corresponding bins from the first and second half of a single trial, using only those bins in which firing rate > 0Hz in at least one half of the trial. The effects of age and environment on intra-trial stability and the RV of HD cells were tested initially by a 2-way ANOVA, followed by tests of simple main effects to assess differences between environments at particular ages.

#### Temporal and Spatial Relationships between Cells

Temporal cross-correlograms were defined as the cross-correlograms (constructed using the MATLAB ‘xcorr’ function) between time-binned histograms of the spike times of two neurons (bin width = 200ms). In order to standardize the height of the central peak (or depth of the central trough) and allow comparisons across all cell pairs, raw temporal cross-correlograms were normalized by the mean of a population of cross-correlograms (100 per pair) constructed with spike-shifted data. The 100 spike-shifted correlograms were constructed by shifting the spikes of one neuron along a set of linearly spaced intervals from 20sec to [trial duration - 20sec]. The mean normalized spike count of the central 1 s of the cross-correlogram was then used as a measure of the degree of offset (i.e., coupled or anti-coupled firing) between neurons. The degree of similarity between central 1 s spike counts in the large and small boxes was compared using linear regression. Only cell pairs in which both contributing cells were classified as HD cells in the small box, and neither were classified as HD cells in the standard box, were included in this analysis.

Time-windowed spatial cross-correlograms between pairs of neurons were constructed analogously to those in [45, 46] but with spatial firing being assessed with respect to head direction, rather than to 2-dimensional space. The spike times of one neuron (the ‘reference’ neuron) were used to define a series of 10 s time windows, each starting at the time of a reference neuron spike (head directions of ‘reference neuron’ were assigned a zero degrees value - windows were reduced in duration if a reference spike occurred within 10 s of the beginning or end of the trial). Within each window, a histogram was created of the head directions associated with the spiking of the second neuron (the ‘test’ neuron; histogram bin width = 6°), with directions being defined relative to the window center. The histograms of all windows were then summed, and smoothed (30° boxcar filter), producing an overall map of the directions in which the test neuron fired relative to the reference neuron, within a short time window. To control for uneven patterns of head rotation, the spike histogram was then divided by a summed, smoothed, histogram of all of the relative directional dwell times across all time windows: this defined the spatial cross-correlogram. To assess the degree of spatially coupled firing and the angle of directional offset, the spatial cross-correlogram (range −180° to 180° from the reference spike) was used to construct a polar rate map (analogous to that for normal head-directional firing), and the mean direction and mean resultant vector length (i.e., Rayleigh Vector) for this map were calculated. A large Rayleigh Vector indicates a strong spatial coupling over the time frame defined by the window. The degree of similarity between angular offsets in the standard and small boxes was compared using circular-circular regression. Only cell pairs for which both contributing cells were classified as HD cells in the small box, and neither were classified as HD cells in the standard box, were included in this analysis.

#### Bayesian Decoding

Decoding in the standard box was performed using standard Bayesian methods (see for example [12, 47]) barring the key difference that the function of a neuron’s average firing with respect to direction (*f*_i_(**x**) in Equation 35, ref [7]), was derived from firing rate maps recorded in the small box trial. The final Bayesian estimate of position:

P(dir|spk)=P(spk|dir)∗P(dir)

was therefore an estimate of the direction which current HD network activity was signaling, defined within the directional reference frame of the small box trial. See Figure S1 for a comparison of this approach with the standard method, decoding standard box position using *f*_i_(**x**) based on standard box rate maps. Only ensembles containing at least 9 HD cells in at least one small box trial were included (N = 6 ensembles). The decoding window was 1 s long, and non-overlapping windows were used. To estimate the angular velocity at which the decoded direction was moving, the decoded position was up-sampled to 50Hz, smoothed with a 5 s Gaussian kernel, and the angular velocity for each sample was estimated as the difference in direction at sample N and sample N+100 (i.e., across a 2 s time step). To produce a comparable estimate of actual head angular velocity (in order to correlate this with the decoded estimate), the actual head direction of the animal was averaged across 1 s long non-overlapping bins (mimicking the output of the decoding algorithm), then up-sampled and smoothed equivalently. The angular velocity was then estimated across 2 s time steps. For small box decoding *f*_i_(**x**) was based on rate maps from the small box trial. In this case, odd minutes of the trial were used to decode even minutes of observed spiking, and vice versa, such that at no point was spiking data used to decode itself. The relationship between actual and decoded angular velocity was tested using linear regression, and the difference between the slopes of these regression fits (between small and standard box) was tested using Student’s t, analogously to testing for the differences between two means [48].

#### Estimation of coupled cell pairs in P12 data

To investigate the presence of temporally or spatially coupled neurons in P12 ensembles, we measured the proportions of temporal cross-correlograms with high or low central 1 s mean normalized correlation (indicating temporally coupled or anti-coupled firing, respectively) and spatial cross-correlograms with high RV scores (indicating consistent spatial firing offsets with the time-window). We defined 95% confidence limits for the chances of finding high or low coupling scores in non-HD cell data, by measuring the 5^th^ and 95^th^ percentiles of the distributions of scores in the population of all known nonHD-nonHD cell pairs that were recorded (N Pair = 3,634). This population was based on all pairs of recorded neurons that were not classified as HD cells, under experimental conditions when stable HD cells could be detected (small box at ages P13-14; standard box and small box at ages P15-P21. The over-representation of coupling scores beyond these limits in P12 data (significantly greater than 5%) was tested using a 1-sample Z-test for proportions.

## Supplemental Information

Document S1. Figures S1–S4

Document S2. Article plus Supplemental Information
