# Supplementary material for: Self-Organized Attractor Dynamics in the Developing Head Direction Circuit
Source: Curr Biol. 2018 Feb 19;28(4):609–615.e3. doi: 10.1016/j.cub.2018.01.010 (PMC5835142; doi:10.1016/j.cub.2018.01.010)
Supplement: Document S1. Figures S1–S4 [file mmc1.pdf]

Current Biology, Volume *28*

Supplemental Information

Self-Organized Attractor Dynamics in the Developing Head Direction Circuit

Joshua P. Bassett, Thomas J. Wills, and Francesca Cacucci

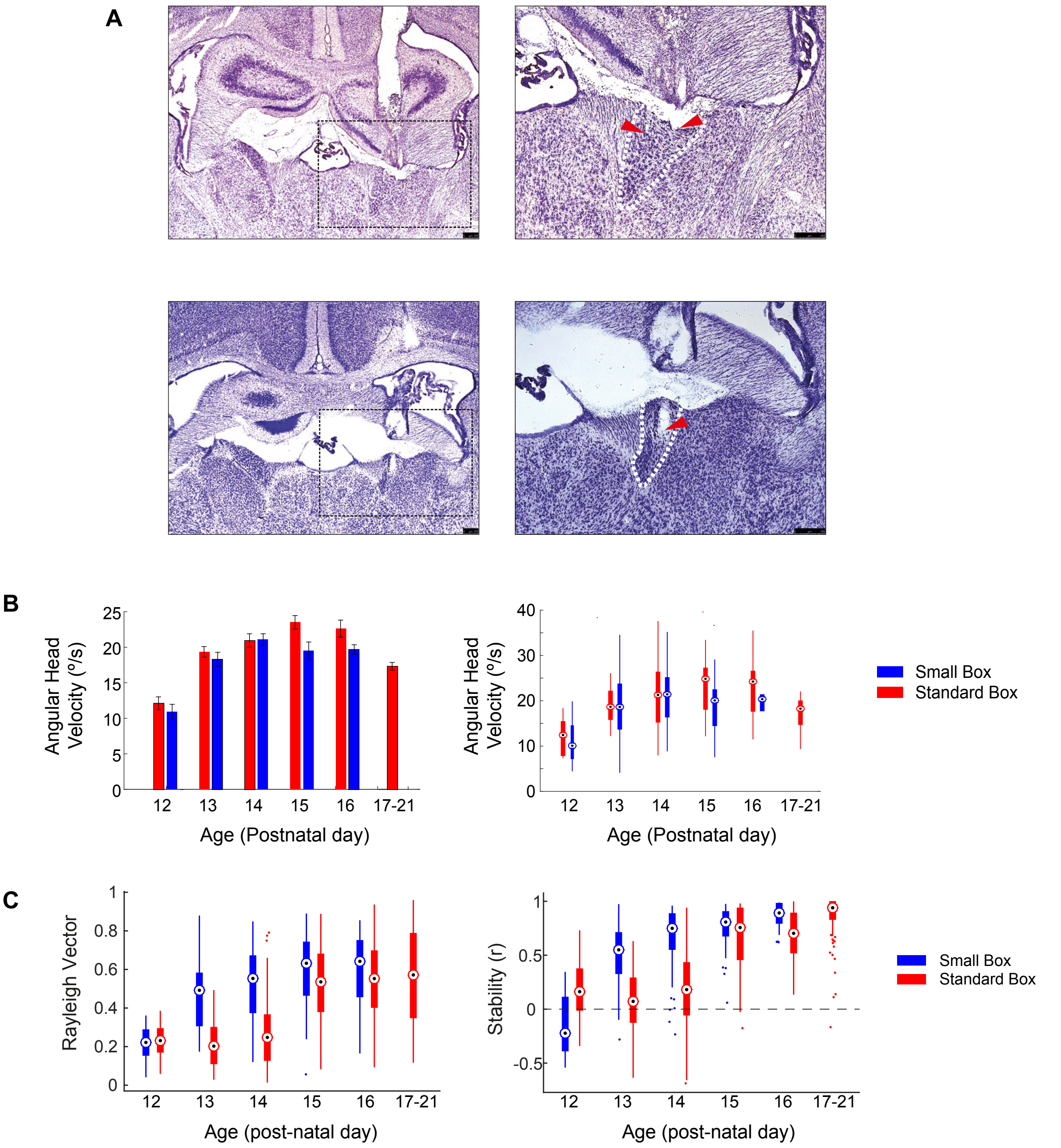

Figure S1. Anatomical recording locations, angular head velocity profiles and box-plots of head direction cell spatial firing properties, Related to Figure 1. (**A**) Nissl-stained brain sections (top, cresyl violet; bottom, thionin) showing representative recording locations in the Anterodorsal Nucleus of the thalamus (ADN). Boxes inset on right (black dashed lines) correspond to area of view on right. Red arrows mark the deepest location through which identifiable tetrode tracks passed. Approximate borders of ADN are outlined in white dashed lines in higher magnification pictures on right. Scale bars represent 250µm. (**B**) Absolute angular head velocity (AHV) does not differ significantly between the small and standard boxes, between P12 and P16. Left: bar chart shows the overall mean (±SEM) of the trial median AHVs in standard and small box, at each age. No significant differences between AHV in small and large box at any age can be detected (2-way ANOVA Age*Env; Env $F_{1,294}$=2.86, p=0.09, Age $F_{4,294}$=21.8, p<0.0001, Env*Age $F_{4,294}$=1.47, p=0.21). Right: the same data as panel B left, but displayed as box-and-whisker plots. For each group, black circle shows median, thick bar shows inter-quartile range (IQR) and thin bar shows the extent of the data, excluding outliers, which are shown as individual data points. Outliers were defined as lying further than 1.5*IQR beyond the nearest quartile. (**C**) Box-and-whisker plots showing distributions of Rayleigh Vector length and intra-trial stability for HD cells recorded in small and standard boxes P12 - P21 (same data as Figure 1B, middle, bottom). Format of box-and-whisker plots is as described in (B).

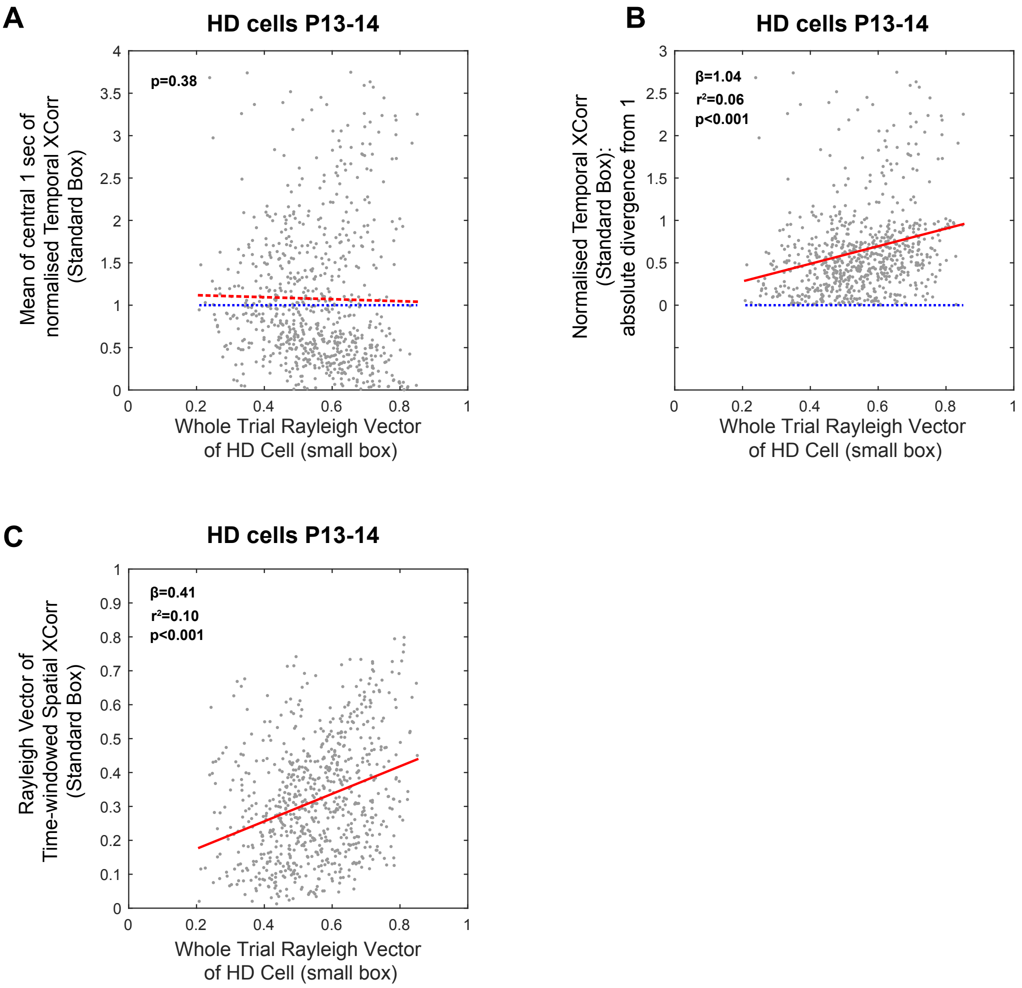

Figure S2. Relationship between head direction cell spatial tuning over a whole trial, and short time-scale temporal/spatial coupling scores, Related to Figure 3. Cross-correlogram scores indicating directionally coupled ADN cells in P12 rats (see Figure 3) cannot be explained by broader directional tuning of HD cells at younger ages. (**A**) Scatter plot showing the signed temporal coupling of known HD cell pairs, when drifting in the standard box at P13-14, versus the mean directional tuning of the cells over a whole trial, when stable in the small box (mean Rayleigh Vector length of directional rate maps; RV). There is no significant correlation between temporal coupling and RV ($p=0.38$, $\beta = -0.12$, $r^2=0.003$). (**B**) Temporal coupling scores diverge from 1 (indicating chance levels of coupling; blue dashed line) as whole trial RV increases, either increasing (indicating temporal co-firing) or decreasing (indicating temporal anti-coupling). We therefore also analysed the unsigned (absolute) temporal coupling of P13-14 HD pairs in standard box vs mean directional tuning of the cells in small box: there is a significant correlation between RV and divergence from a temporal coupling score of 1 (see Figure for stats), though the amount of variance explained by RV is low ($r^2 = 0.06$). (**C**) Scatter plot showing time-windowed spatial coupling of known HD cell pairs, drifting in the standard box at P13-14, versus the mean directional tuning of the cells over the whole trial in the small box (RV). Spatial coupling scores increase as mean RV increases (linear regression; see Figure for stats). Hence, the lower RV of HD cells seen in young rats cannot lead to artefactual over-estimation of temporal and spatial coupling scores at P12. We cannot rule out that the temporal and spatial cross-correlogram scores are under-estimating the numbers of directionally coupled cells at this age.

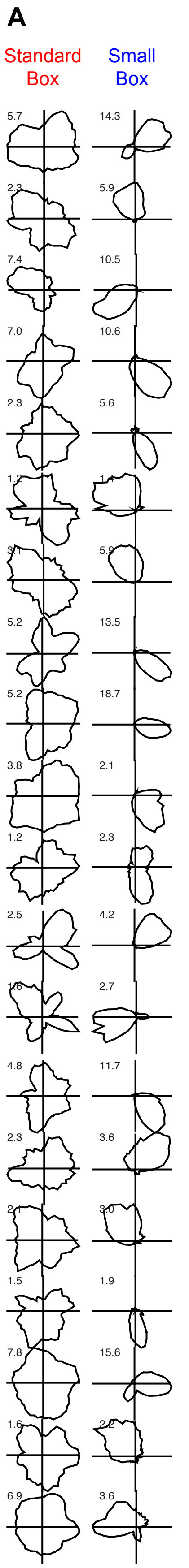

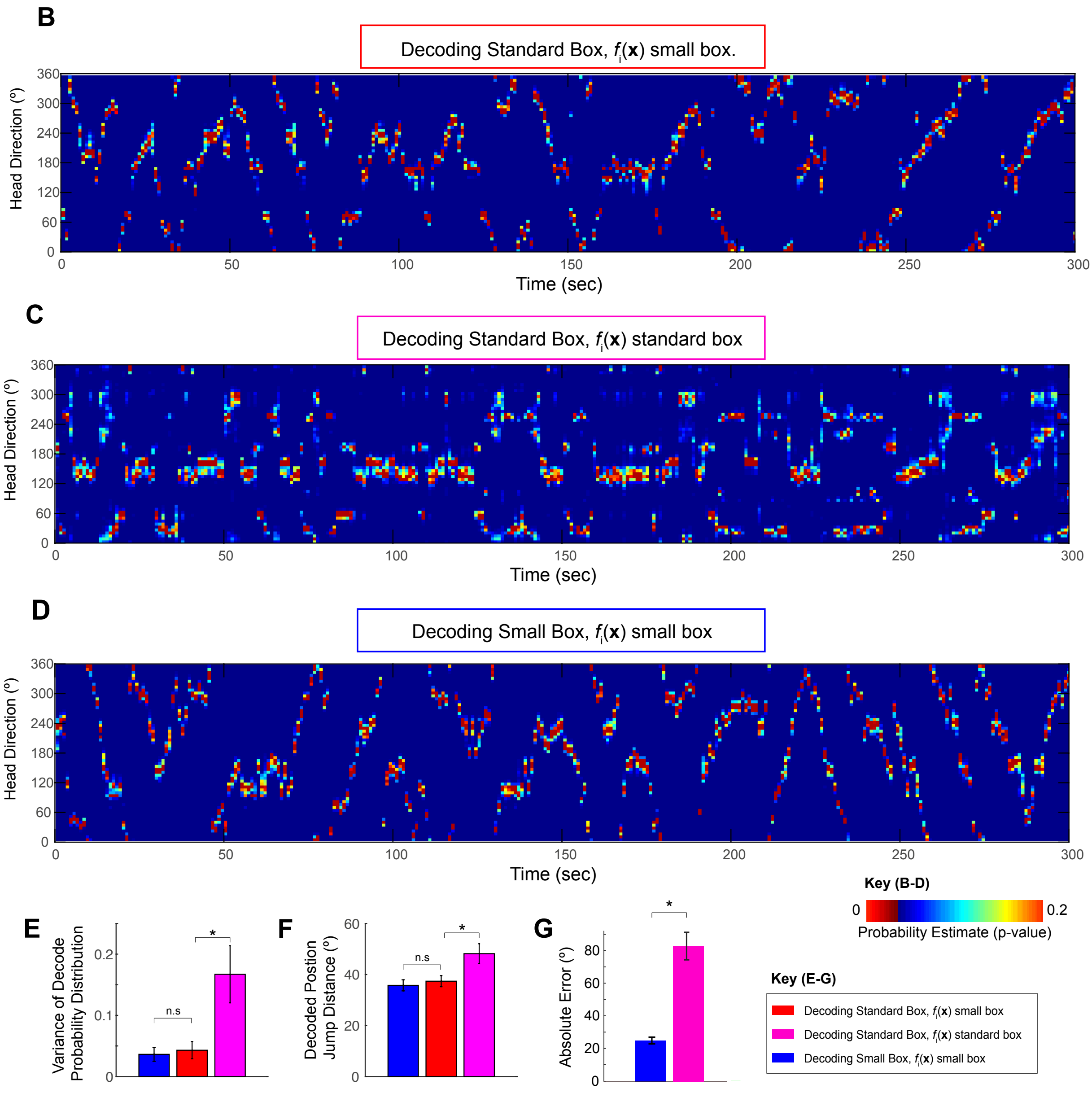

Figure S3. **Validation of cross-trial (small/standard box) Bayesian decoding approach at P13-P14, Related to Figure 4.** (**A**) Polar plots for all HD cells used for decoding in the example of Figure 2A, recorded in standard box and small box (P14). HD cells show stable directional signalling in the small box (right), but not in the standard box (left). (**B**) Full set of decoded probability estimates for example shown in Figure 2A. False colour image showing probability estimates of the rat facing in each direction (y-axis, bin size 6° width), computed from HD spiking in each decoding window (x-axis, window size = 1 sec). The function of average firing in a given direction for each cell ($f_i(x)$ where i represents the cell and x the direction) was based on polar plots derived from the small box trial. A coherent cluster of high probability is apparent in each decoding window and the estimated direction (maximum probability) moves smoothly between time bins, consistent with the presence of continuous attractor dynamics in the HD cell network, even though the HD network is not anchored to laboratory reference frame (see polar plots in (A) left column). (**C**) Full set of probability estimates obtained by decoding the same data as (A) and Figure 2A, in the case where $f_i(x)$ is based on the polar plots derived from the standard box trial. Consistent with the reduced spatial tuning and stability of the polar plots in the standard box, distributions of probability estimates within each decoding window display a high variance, and discontinuous jumps between temporally contiguous decoding windows are apparent. (**D**) 5 minute example of full probability estimates obtained decoding the small box trial (polar plots in (A), rightmost column). $f_i(x)$ was based on data derived from the same small box trial data (interleaved 60-sec time epochs were used for $f_i(x)$ construction and decoding, respectively, see methods for details). Comparison with (B) shows that the circular coherence of the probability estimates and the smoothness of the decoded trajectory are qualitatively similar whether decoding the small box, or the standard box using small box $f_i(x)$. (**E**) For all decoded ensembles (N=6), there is no significant difference in the circular coherence of probability within each window (measured by weighted circular variance), when comparing small box decoding and standard box decoding using small box $f_i(x)$. By contrast, standard box decoding using standard box $f_i(x)$ leads to significantly greater variance (ANOVA; $F_{2,15}$=6.51, p=0.009; Post-hoc HSD; small vs standard $f_i(x)$ small, p=0.98, standard $f_i(x)$ small vs $f_i(x)$ standard, p=0.021). (**F**) For all decoded ensembles, there is no significant difference in the smoothness of decoded trajectories (measured by the mean angular jump between consecutive decoded directions), when comparing small box decoding and standard box decoding using small box $f_i(x)$. By contrast, standard box decoding using standard box $f_i(x)$ leads to significantly greater average jumps (ANOVA; $F_{2,15}$=5.6, p=0.015; Post-hoc HSD; small vs standard $f_i(x)$ small, p=0.91, standard $f_i(x)$ small vs $f_i(x)$ standard, p=0.043). (**G**) Spatial accuracy of decoding, measured by absolute distance between actual and decoded head direction. Decoding the small box leads to absolute errors consistent with previously published studies[14]. By contrast, absolute error in the standard box is larger when using small box $f_i(x)$, indicating that the HD cells are not stably anchored to the testing environment reference frame (T-Test, t_5=6.1, p=0.002).

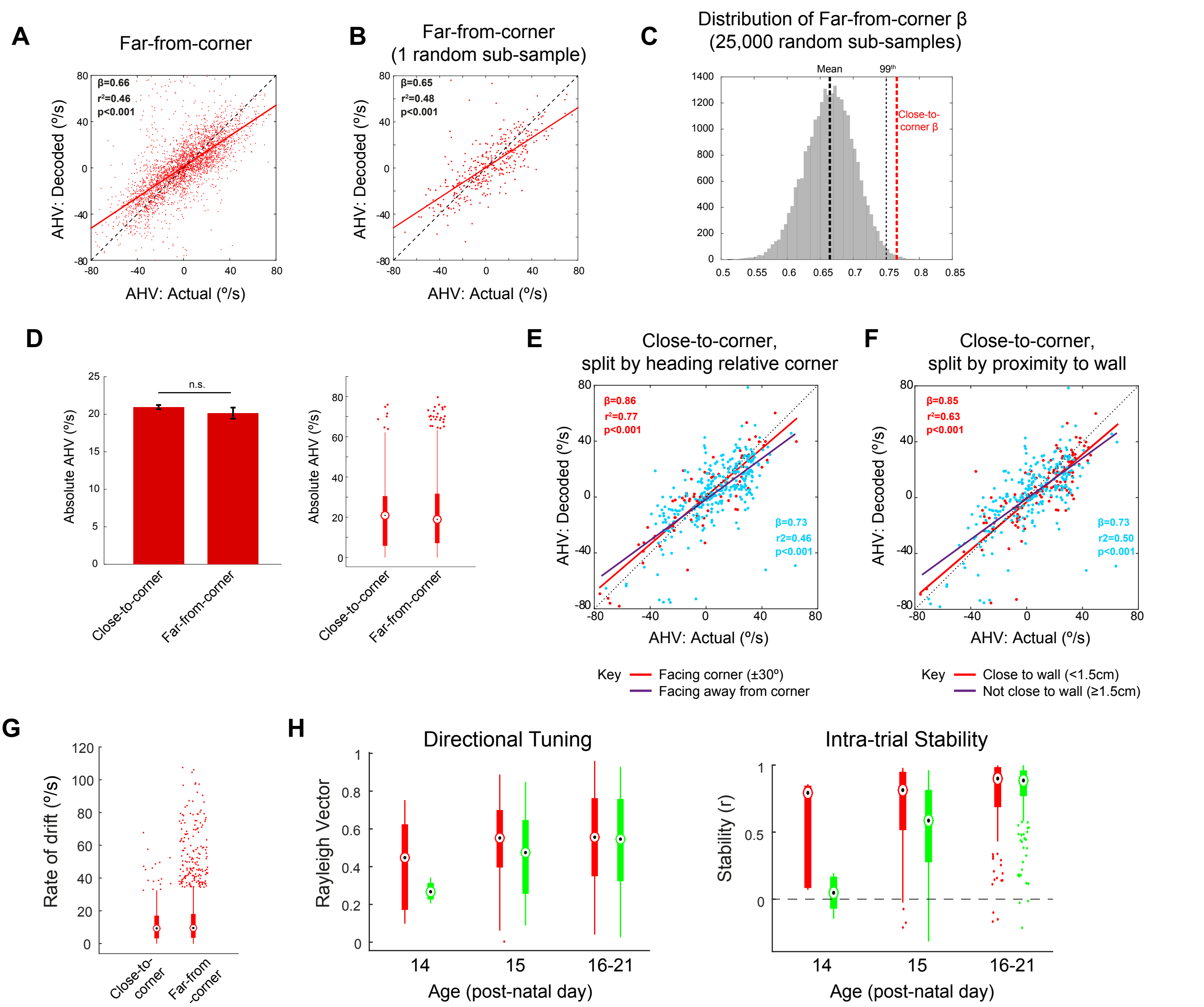

Figure S4. Control analyses for mitigation of angular head velocity (AHV) under-signalling close to corners, evidence for further mitigation of AHV under-signalling when facing corners, box-plots of rate of drift, and spatial properties of head direction cells in the circular environment, Related to Figure 4. (**A-C**) The difference between real versus decoded angular head velocity (AHV) regression slopes, for data recorded close-to-corners as compared to far-from-corners, is not due to different numbers of samples in the two groups. (**A**) Real versus decoded AHV regression for far-from-corner (same as Figure 4B, right). (**B**) The same data as (A), but randomly sub-sampled to have the same number of data points as the close-to-corner group (N=391). The regression slope is similar to the full dataset. (**C**) Distribution of regression slopes for 25,000 random sub-samples (to N=391) of the far-from-corner dataset. The mean regression slope of the sub-samples (0.667; heavy black dashed line) is very similar to that of the full dataset. The slope of the close-to-corner full dataset (0.774; red dashed line) lies beyond the 99th percentile of the sub-sampled slope distribution (light dashed black line). (**D**) Absolute AHV does not differ between close-to- and far-from-corner data. Right: bar chart shows mean (±SEM) AHV for each group. Means are not significantly different (t-test: $t_{(3478)}$=0.97, p=0.33). Left, box-and-whisker plot for the same data. For each group, black circle shows median, thick bar shows inter-quartile range (IQR) and thin bar shows the extent of the data, excluding outliers, which are shown as individual data points. Outliers were defined as lying further than 1.5*IQR beyond the nearest quartile. (**E-F**) AHV under-signalling is further reduced when rats are both close to corners, and (E) either facing the corner or (F) in very close proximity to the wall, suggesting that physical contact with the wall may help stabilise the HD signal. (**E**) Real versus decoded AHV linear regression for close-to-corner data, split according to whether the rat is facing the corner (looking directly at corner ±30°; red) or facing away from corner (sky blue). The facing corner regression slope is smaller than the non-facing slope, approaching significance at the p=0.05 level (t-test: $t_{(387)}$=1.593, p=0.054). The difference in slopes is unlikely due to the differences in sample size: when the facing away group data was randomly subsampled to match the facing group N (83), the actual slope for the facing corner group (β=0.862) was greater than the 90th percentile of the subsampled regression slopes (β=0.856). (**F**) Real versus decoded AHV linear regression for close-to-corner data, split according to whether the rat is in close proximity to a wall (when tracking lights on head are <1.5cm from wall; red) or further from a wall (sky blue). The close to wall regression slope is smaller than the further from wall slope, approaching significance at the p=0.05 level (t-test: $t_{(387)}$=1.631, p=0.051). The difference in slopes is unlikely due to the differences in sample size: when the facing away group data was randomly subsampled to match the facing group N (125), the actual slope for the facing corner group (β=0.854) is greater than the 90th percentile of the subsampled regression slopes (β=0.840). (**G**) Box-and-whisker plots for distributions of rate of drift, for data recorded close-to- and far-from-corners (same data as Figure 4C). Format of box-and-whisker plots is as described in (D). (**H**) Box-and-whisker plots for distributions of directional tuning (left) and intra-trial stability (right) of HD cells recorded in the standard box (red) and circle (green). (Same data as for Figure 4E). Format of box-and-whisker plots is as described in (D). There was an increase in the variability of the directional tuning of HD cells in the circle between P14 and P15 (F-test for RV: $F_{(4,98)}$=0.532, p=0.011), indicating that, at P14, only poorly tuned responses were observed, whereas by P15 both strong and poor directional tunings were observed. No other significant differences between group variances were observed (F-test, all p<0.1).
