# Supplementary material for: Self-Organized Attractor Dynamics in the Developing Head Direction Circuit
Source: Curr Biol. 2018 Feb 19;28(4):609–615.e3. doi: 10.1016/j.cub.2018.01.010 (PMC5835142; doi:10.1016/j.cub.2018.01.010)
Supplement: Document S2. Article plus Supplemental Information [file mmc2.pdf]

Report

Current Biology

Self-Organized Attractor Dynamics in the Developing Head Direction Circuit

Highlights

- Non-visual cues can anchor head direction (HD) cells in pre-eye-opening rat pups
- Internal network dynamics are preserved even when the HD representation is unstable
- Angular velocity under-signaling drives instability, which is mitigated by corners
- Circuit architecture develops even before any landmarks can stabilize HD responses

Authors

Joshua P. Bassett, Thomas J. Wills, Francesca Cacucci

Correspondence

t.wills@ucl.ac.uk (T.J.W.),
f.cacucci@ucl.ac.uk (F.C.)

In Brief

Bassett et al. show that the attractor architecture of the head direction (HD) circuit develops without reference to landmarks. Non-visual cues can stabilize HD responses in rat pups aged 13 days or older. Instability in pre-visual HD cells is caused by angular velocity under-signaling, mitigated by proximity to corners in square enclosures.

Bassett et al., 2018, Current Biology *28*, 609–615
February 19, 2018 © 2018 The Author(s). Published by Elsevier Ltd.
https://doi.org/10.1016/j.cub.2018.01.010

CellPress

Current Biology
Report

CellPress

Self-Organized Attractor Dynamics in the Developing Head Direction Circuit

Joshua P. Bassett,[1] Thomas J. Wills,[2,3,*] and Francesca Cacucci[1,3,4,*]
[1]Department of Neuroscience, Physiology and Pharmacology
[2]Department of Cell and Developmental Biology
University College London, Gower Street, London WC1E 6BT, UK
[3]Senior author
[4]Lead Contact
*Correspondence: t.wills@ucl.ac.uk (T.J.W.), f.cacucci@ucl.ac.uk (F.C.)
https://doi.org/10.1016/j.cub.2018.01.010

SUMMARY

Head direction (HD) cells are neurons found in an extended cortical and subcortical network that signal the orientation of an animal's head relative to its environment [1–3]. They are a fundamental component of the wider circuit of spatially responsive hippocampal formation neurons that make up the neural cognitive map of space [4]. During post-natal development, HD cells are the first among spatially modulated neurons in the hippocampal circuit to exhibit mature firing properties [5, 6], but before eye opening, HD cell responses in rat pups have low directional information and are directionally unstable [7, 8]. Using Bayesian decoding of HD cell ensemble activity recorded in the anterodorsal thalamic nucleus (ADN), we characterize this instability and identify its source: under-signaling of angular head velocity, which incompletely shifts the directional signal in proportion to head turns. We find evidence that geometric cues (the corners of a square environment) can be used to mitigate this under-signaling and, thereby, stabilize the directional signal even before eye opening. Crucially, even when directional firing cannot be stabilized, ensembles of unstable HD cells show short-timescale (1–10 s) temporal and spatial couplings consistent with an adult-like HD network. The HD network is widely modeled as a continuous attractor whose output is one coherent activity peak, updated during movement by angular head velocity signals and anchored by landmark cues [9–11]. Our findings present strong evidence for this model, and they demonstrate that the required network circuitry is in place and functional early during development, independent of reference to landmark information.

RESULTS AND DISCUSSION

Local Cues Can Stabilize Developing HD Networks from Post-natal Day 13 Onward

Previous studies recorded head direction (HD) cells from rat pups moving within a 62 × 62-cm open-field environment (standard box) typical of that used with adult animals [5, 7]. Here we also introduced animals to a second smaller environment (small box, 20 × 20 cm). We recorded anterodorsal thalamic nucleus (ADN) neurons (1,276 cells from 21 animals, aged post-natal day (P)12–P21; see Figure S1A for anatomical location of recording sites) while rat pups alternately explored the two environments (Figure 1A). The proportion, spatial tuning, and stability of HD cells were higher in the small versus the standard box (Figures 1B and S1C), most markedly at the pre-visual ages P13–P14 (2-sample Z-test for proportions, $Z > 7.5$, $p < 0.0001$ for both P13–P14; ANOVA Rayleigh vector [RV], Age*Env $F_{4,1025} = 15$, $p < 0.001$, simple main effects $(SME)_{(ENV)}$ $p < 0.001$ at P13–P14; ANOVA stability, Age*Env $F_{4,1019} = 45$, $p < 0.001$, $SME_{(ENV)}$ $p < 0.001$ at P13–P14). There was no difference between the proportions of neurons classified as HD cells in the small box at P13 and those at P17–P21, when the HD system was fully mature ($Z = 1.1$, $p = 0.27$). Sensory modalities other than vision are, therefore, capable of anchoring HD representations before eye opening. Angular head velocity did not differ significantly between environments, excluding this as cause for increased stability in the small box (Figure S1B).

At P13–P14, the internal organization of the HD network appeared to be preserved even when HD responses were unanchored to the environmental reference frame (standard box): for each head turn direction, similar spiking sequences could be observed across co-recorded HD cells in both small and standard boxes. Network activity transitioned smoothly through directional space, while often undershooting actual head movements (Figure 1C).

HD Cells in Developing Rats Display Adult-like Spatial and Temporal Coupling, Even When Drifting

To confirm whether the internal organization of the HD network is preserved, even when its responses are unanchored from the external environment, we examined the short-term temporal and spatial couplings between pairs of co-recorded neurons. We computed temporal cross-correlograms (Figures 2A and 2B) and time-windowed spatial cross-correlograms (Figures 2D and 2E) for all pairs of co-recorded cells that displayed HD tuning in the small box (see the STAR Methods). To eliminate residual HD stability in the standard box as a confounding factor, we excluded any cells that displayed significant HD tuning in the standard box. Despite this, both the temporal and spatial relationships between pairs of co-recorded HD cells were preserved across both the small and standard environments

© 2018 The Author(s). Published by Elsevier Ltd.
This is an open access article under the CC BY license (http://creativecommons.org/licenses/by/4.0/).

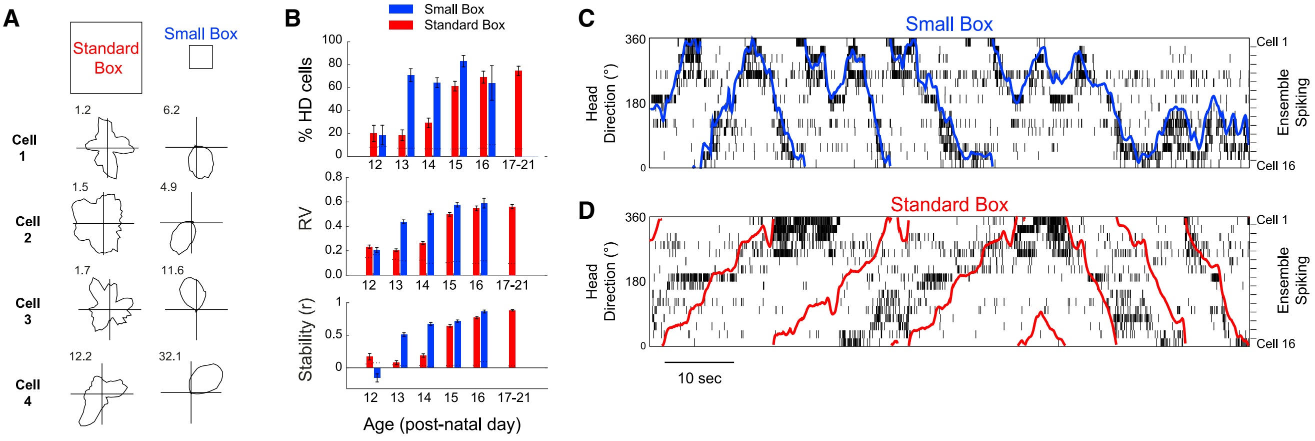

Figure 1. HD Cell Signaling Is Stabilized When Rats Explore a Small (20 cm Side) Environment, from P13 Onward

(A) Example firing rate polar plots for 4 HD cells recorded at P13 in the standard (left) and small (right) boxes. Numbers at top left indicate peak firing rate (Hz).

(B) HD cells are more numerous (top) and have a higher spatial tuning (Rayleigh vector [RV], middle) and intra-trial stability (bottom) when recorded in a small versus a standard box in animals older than P13. Error bars indicate SEM. See also Figure S1.

(C and D) HD cell network internal organization is preserved in the standard box, even when directional signaling is unstable. Colored traces indicate the rat's actual head direction, overlaid upon spike raster plots for all simultaneously recorded HD cells, in the small (C) and standard (D) boxes. For both (C) and (D), HD cells are ordered vertically by their preferred firing direction in the small box. The sequences of HD cell activation for each head turn direction are similar in the small and standard boxes, but the direction signaled by HD cell firing consistently undershoots actual rotation in the standard box.

(significantly correlated temporal and spatial offsets between small and standard boxes, see Figures 2C and 2F). This demonstrates that, even when HD cells are unstable in the open field (on P13–P14), the internal network structure is unchanged.

Attractor Connectivity Precedes HD Landmark Stabilization

Introducing the youngest group of animals (P12) to the small box did not result in an improvement in HD cell stability/tuning (Z-test for %HD, $Z = 0.71$, $p = 0.45$; $SME_{(ENV)}$ for RV, $p = 0.99$; for stability, small box lower; Figure 1B). Nevertheless, many co-recorded cell pairs showed temporal and spatial coupling indicative of an attractor neural structure, even at this age (Figures 3A–3C). To test whether the degree of temporal and/or spatial coupling at P12 was significantly higher than chance, we measured the proportions of P12 cross-correlogram scores lying beyond 95% confidence limits for HD-HD coupling (defined as the fifth and 95th percentiles of the scores from all known non-HD cell pairs in older rats; Figure 3D). The proportion of P12 cell pairs with coupling scores beyond these confidence limits significantly exceeded 5% (one sample Z-test; temporal, $Z = 19$, $p < 0.001$ [fifth], $Z = 14$, $p < 0.001$ [95th]; spatial, $Z = 33$, $p < 0.001$), demonstrating that HD neurons display fixed temporal and spatial offsets at P12, an age at which none of the tested experimental manipulations resulted in environmentally stable HD responses. The presence of these temporal and spatial couplings cannot be explained by the overall broader tuning of HD cells at younger ages (Figure S2).The HD network thus displays a key signature of continuous attractor structure before HD responses can be stabilized by local landmarks.

Drift of the HD Representations Occurs due to Systematic Under-Signaling of Angular Head Velocity

To further characterize the temporal dynamics of drifting HD networks, we applied a cross-trial Bayesian decoding approach, using firing rate maps of stable HD cells in the small box to decode signaled direction (in small box coordinates, see the STAR Methods) as the rat moved in the standard box, at P13–P14. Although, as expected, decoded headings diverged from actual headings, decoding produced a coherent estimate of direction exhibiting continuous, smooth transitions during a trial, consistent with attractor dynamics (Figure 4A, top; Figure S3). The coherence and smoothness of decoded trajectories were not significantly different whether decoding was performed on standard or small box data, further demonstrating the maturity of the internal network dynamics, despite ongoing drift (Figures S3E and S3F). We then obtained the decoded angular head velocity by calculating the first derivative of the decoded head direction (angular head velocity, Figure 4A, bottom; see the STAR Methods). We found that, when HD responses are drifting, although angular head velocity is linearly correlated with actual head velocity, it is under-signaled by drifting HD networks in the standard box (Figure 4B, regression β significantly lower for standard box, $t_{(6916)} = 16.54$, $p < 0.0001$). Thus, the most prominent source of error in young HD networks is a systematic under-signaling of angular head velocity, which cannot be compensated for by alternative sensory cues in the standard box.

Geometric Cues Can Mitigate Path Integration Error

Introducing rat pups in the small box results in stabilization of HD responses (Figures 1A, 1B, and S1C) and reduction of angular head velocity under-signaling (Figure 4B, left). Following previous findings that boundaries stabilize place fields during post-natal development [12] and grid cell signaling in adult mice [13], we tested whether HD cells were more stable when animals were close to an environmental boundary. Unexpectedly, the rate of drift (rate of divergence between actual and decoded heading) was significantly greater close to walls (mean rate of drift: walls = 13.3 ± 0.52 degrees/s, center = 12.2 ± 0.70

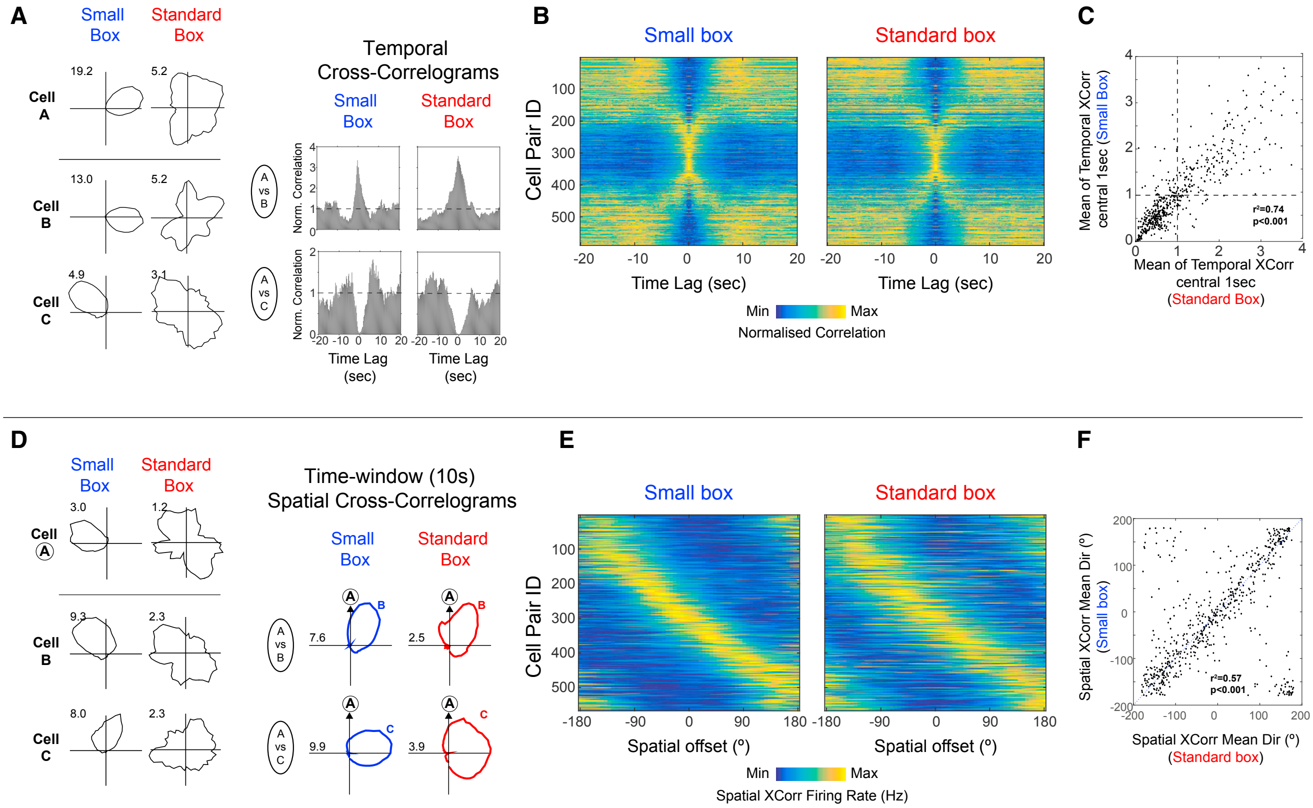

Figure 2. Short-Timescale Temporal and Spatial Couplings between HD Cells Are Preserved Even When Directional Signaling Is Unstable

(A) Example polar plots (left) and temporal cross-correlograms (TXCs, right) for 3 HD cells recorded in small and standard boxes.

(B) TXCs of all co-recorded HD cell pairs in the small (left) and standard (right) boxes, normalized between their minimum (dark blue) and maximum (yellow) values. Each row in the image shows one TXC, and rows are sorted on the basis of preferred firing direction difference in the small box.

(C) Correlation between mean values of the central 1 s of TXCs in the small versus standard box.

(D) Example polar plots (left) and time-windowed (10 s) spatial cross-correlograms (SXCs, right) for 3 HD cells.

(E) SXCs of all co-recorded HD cell pairs in the small (left) and standard (right) boxes. HD pairs are sorted as in (D).

(F) Circular-circular correlation between the mean directions of SXCs in the small versus standard box.

degrees/s; $t_{(3476)} = 2.5$, $p = 0.007$). This is likely caused by an increase in actual angular head velocity when animals are close to walls (mean angular head velocity: walls = 21.6 ± 0.6 degrees/s, center = 20.2 ± 0.8 degrees/s; $t_{(3476)} = 2.70$, $p = 0.012$). However, further analyses showed that both the rate of drift and angular head velocity under-signaling were significantly reduced when animals were close to a corner (mean rate of drift: $t_{(3476)} = 1.98$, $p = 0.048$; see Figures 4C and S4G; angular head velocity under-signaling difference of β: $t_{(3476)} = 2.47$, $p = 0.013$; see Figure 4D). This effect cannot be accounted for by differences in the number of samples or differences in actual angular head velocity close to and far from corners (Figures S4A–S4D). Additional evidence suggests that angular head velocity under-signaling is further reduced when rats are close to corners and either face the corner or have their head in close proximity to the wall (Figures S4E and S4F). Corners may, therefore, be a geometric feature used to offset path integration error in developing animals.

To further investigate the stabilizing influence of corners on the developing HD network, we introduced rat pups (P14–P15) to a circular environment of similar dimensions to the standard square environment (matched perimeter). When recorded in the circular environment, HD cells showed reduced directional tuning and intra-trial stability (Figures 4E and S4H; directional tuning [RV]: ANOVA Age*Env $F_{(2,348)} = 8.5$, $p < 0.001$; $SME_{(ENV)}$: P14, $p = 0.023$; P15, $p < 0.001$; stability: ANOVA Age*Env $F_{(2,343)} = 18.9$, $p < 0.001$; $SME_{(ENV)}$: P14, $p < 0.001$; P15, $p < 0.001$). These findings further support the interpretation that corners may be used to stabilize HD cells in early development.

A striking characteristic of HD cells is that, although their *absolute* preferred firing directions can change (following disorientation or manipulations of the environment), the tunings of simultaneously recorded cells retain their *relative* spatial offsets under all circumstances [14, 15]. This experimental finding underlies all available models of the HD circuit, describing it as possessing 1-dimensional ring architecture, with the connectivity between HD units arranged such that nodes with similar heading preferences are mutually excited, whereas nodes with divergent preferred directions inhibit each other [9–11]. This topology gives rise to continuous attractor dynamics, whereby the network always produces a single, localized peak of activity involving neighboring nodes, which represents the current directional bearing. In adult mice, HD cell firing

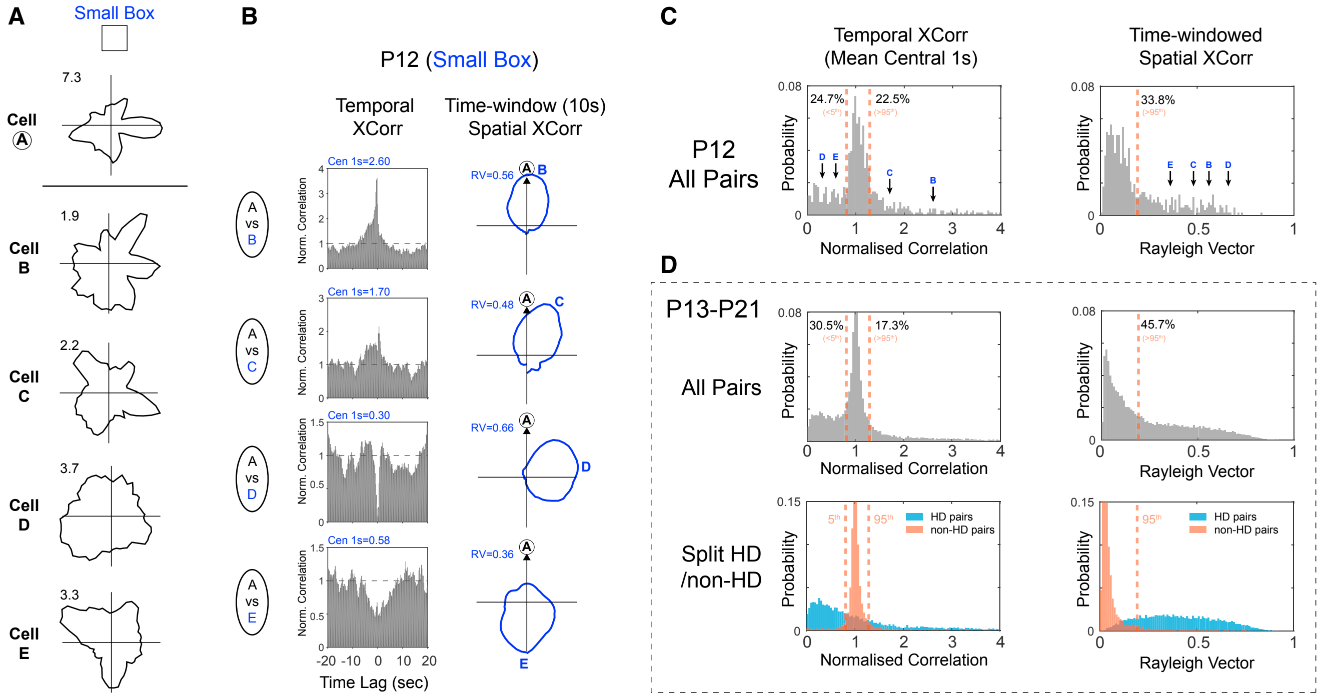

Figure 3. HD Attractor Network Organization Is Present at P12, Even before HD Cells Can Be Anchored to Landmarks

(A) Polar plots for five example cells recorded in a small box at P12, showing no HD tuning over a 10-min session.

(B) Temporal cross-correlograms (TXCs; left) and spatial cross-correlograms (SXCs; right) between cell A and cells B–E. Blue text at top left shows mean of central 1 s of TXC and RV length of SXC, respectively, for each example.

(C) Probability distributions of mean of central 1 s of the TXC (left) and RV length of the SXC (right) scores for all P12 co-recorded cell pairs (n = 452). Black arrows with blue letters indicate scores from examples shown in (A) and (B). Orange dashed lines show the values of the fifth and 95th percentiles of scores of known non-HD cells in older animals (see D; only 95th percentile is shown for SXC). Black text refers to percentages of P12 scores above or below these percentiles.

(D) Top row: As for (C) but for all co-recorded cell pairs P13–P21. Bottom row: Same data as top row but distributions of HD-HD pairs (light blue) and nonHD-nonHD pairs (orange) are plotted separately. Orange dashed lines shown here and in (C) and (D) (top row) are derived from the fifth and 95th percentiles of the orange (nonHD-nonHD) distributions. See also Figure S2.

during sleep is consistent with attractor dynamics [16], and HD correlates are preserved during spindles in rats, without movement or landmark input [17]. The position of the activity peak is thought to be updated in response to head movement by the activity of upstream angular velocity-responsive cells, which have been described in the rat midbrain [18, 19]. In this model, the network integrates directional position from velocity, to support the angular component of path integration behaviors. Indeed, lesions of the HD system significantly impair performance on spatial tasks that depend on angular path integration [20, 21].

Path integration is prone to overwhelming accumulation of error without periodic reference to stable external cues, which discharge the error and anchor the spatial representation to the environment [9–11]; if the waking HD system is both an attractor and a path integrator, its development requires not only the intrinsic ring topology but also a self-motion input (e.g., angular head velocity) and landmark cues corresponding to stable features of the environment. Theoretical models of the emergence of the HD system suggest the presence of a distal landmark cue that acts as a supervisory input, simultaneously anchoring the network to the external world and instructing the correct wiring across nodes [22, 23]. Previous studies of HD network development have shown that, even before eye opening, when HD cells exhibit stable tuning within a trial, they also retain their relative spatial offsets between trials, consistent with the behavior of adult HD cells [8]. However, this observation alone cannot address the central question of whether the internal organization of the circuit precedes the emergence of stable directional signaling or rather, as predicted by theoretical models of HD circuit development, these two features of the network arise concomitantly [22, 23].

Here we show that, during early post-natal development (P12–P14) before eye opening, the internal dynamics of the HD circuit, when observed at fine timescales, are exactly as described in a mature continuous attractor network, even while the circuit's spatial relationship to the environment is completely adrift from moment to moment. The internal organization of the HD circuit therefore appears to be in place before its ability to represent heading direction is manifested. Our results provide new constraints on models of continuous attractor network development, as they demonstrate that the rigid coupling of spatial relationships across directional nodes is not extracted from the structure of sensory input through a learning process [22, 23], but instead likely arises through internal, self-organized processes during

CellPress

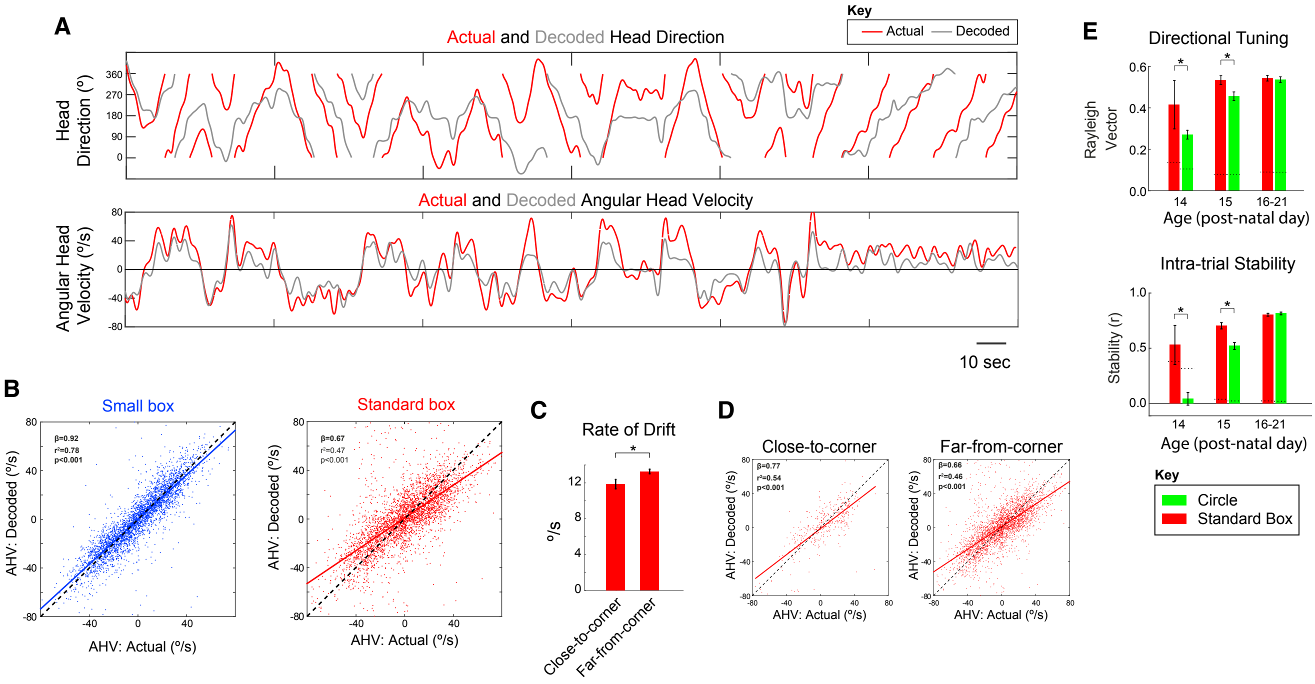

Figure 4. HD Drift in Young Rodents Is Caused by Angular Head Velocity Under-Signaling and Is Reduced by Proximity to Corners

(A) Example of actual (red) and decoded (gray) head direction (top) and angular head velocity (bottom) values displayed by a P14 rat during 5 min exploration in the standard box. See also Figure S3.

(B) Correlation between actual (x axis) and decoded (y axis) angular head velocity scores in the small (left) and standard (right) boxes across all decoded ensembles (n = 6). Slope of relationship between actual and decoded angular head velocity is significantly smaller in the standard versus small box.

(C) Mean (±SEM) rate of drift (rate of divergence between actual and decoded head direction) when rats were close or far from the corners of the standard box.

(D) Angular head velocity under-signaling is reduced when rats are close to corners. Correlations are shown between actual and decoded angular head velocity scores in the standard box, split by proximity to corners (left, close; right, far). See also Figure S4.

(E) Directional tuning and intra-trial stability of HD cells are reduced in a circular environment, as compared to the standard (square) box, on P14–P15. Bar charts show the mean (±SEM) Rayleigh vector (top) or intra-trial stability (bottom) of HD cells recorded in standard and circular environments.

development (with possible further refinement through learning [24]). This finding may also generalize to other putative attractor networks such as grid cells [25–28], which display strong coupling as soon as they can be detected in young animals [29], and the recently discovered neural representation of direction in *Drosophila* [30–33].

HD cells in the ADN reflect the influence of both ascending self-movement information from the tegmento-mammillary circuit and descending visual landmark information from cortical projections [34]. The reciprocal connections between the tegmental and mammillary nuclei as well as the mammillo-thalamic projections are developmentally complete by approximately P10 [35, 36]. Therefore, the generative tegmento-mammillary circuit in which the attractor architecture is thought to be expressed is structurally complete by the time we find attractor dynamics in the ADN, at P12, days before eye opening. Our present findings therefore suggest that the tegmento-mammillary circuit is both necessary for driving HD cell activity in the ADN, in line with the effects of lesions in the adult brain [37], and sufficient without a requirement for supervisory visual input even during its initial organization.

The HD attractor in young animals shows a linear response to angular head velocity but with reduced gain, indicating systematic under-signaling. This constitutes direct experimental evidence that the attractor's activity peak is shifted around the ring proportionally to the magnitude of angular head velocity input [38], thereby integrating velocity to position as predicted by current models of the HD network. In the open field, the under-signaling of velocity, without the ability to correct the accumulating error with landmarks amounts to an open-loop state, resulting in drift of directional firing with respect to the environment. It remains undetermined whether the ultimate cause for this under-signaling can be imputed to reduced vestibular input [39], to its incomplete integration by the HD network, or a combination of the two.

Our results also suggest that geometrical features (corners) can be used to stabilize HD responses during early post-natal development, at a time when access to distal cues is still limited. A possible mechanism for this geometric feature detection, which could additionally explain the time course of its development between P12 and P13, is the progressive refinement of whisking at these ages [40]. An important question for future research is whether geometric input to HD cells is affected by environmental rearing conditions [41]. These results also provide new mechanistic insights as to the recognized importance of geometric features in the development of spatial cognition in children [42, 43].

STAR★METHODS

Detailed methods are provided in the online version of this paper and include the following:

- KEY RESOURCES TABLE
- CONTACT FOR REAGENT AND RESOURCE SHARING
- EXPERIMENTAL MODEL AND SUBJECT DETAILS
- METHOD DETAILS
  - Surgery and electrodes
  - Single-unit recording
  - Behavioral Testing
- QUANTIFICATION AND STATISTICAL ANALYSIS
  - Construction of firing rate maps
  - Classification of single-units as HD cells
  - Quantitative analysis of directional signaling
  - Temporal and Spatial Relationships between Cells
  - Bayesian Decoding
  - Estimation of coupled cell pairs in P12 data

SUPPLEMENTAL INFORMATION

Supplemental Information includes four figures and can be found with this article online at https://doi.org/10.1016/j.cub.2018.01.010.

ACKNOWLEDGMENTS

We acknowledge funding from the ERC (DEVSPACE grant to F.C. and J.P.B. position); the European Union's Horizon 2020 research and innovation programme under grant agreement 720270; the Medical Research Council, UK (MR/N026012/1 to T.J.W.); and the Royal Society (UF150692 to T.J.W.). We would like to thank the following individuals for helpful discussions of previous versions of this paper: Caswell Barry, Andrej Bicanski, Neil Burgess, Ila Fiete, Andreas Herz, Kate Jeffery, Colin Lever, and Hector Page.

AUTHOR CONTRIBUTIONS

J.P.B., F.C., and T.J.W. conceived the experiments, and J.P.B. and F.C. conducted them. T.J.W. analyzed the data with contributions from F.C. and J.P.B. F.C., T.J.W., and J.P.B. wrote the manuscript.

DECLARATION OF INTERESTS

The authors declare no competing interests.

Received: November 14, 2017
Revised: December 13, 2017
Accepted: January 3, 2018
Published: February 1, 2018

REFERENCES

1. Taube, J.S., Muller, R.U., and Ranck, J.B., Jr. (1990). Head-direction cells recorded from the postsubiculum in freely moving rats. I. Description and quantitative analysis. J. Neurosci. *10*, 420–435.
2. Taube, J.S. (2007). The head direction signal: origins and sensory-motor integration. Annu. Rev. Neurosci. *30*, 181–207.
3. Finkelstein, A., Derdikman, D., Rubin, A., Foerster, J.N., Las, L., and Ulanovsky, N. (2015). Three-dimensional head-direction coding in the bat brain. Nature *517*, 159–164.
4. O'Keefe, J., and Nadel, L. (1978). The Hippocampus as a Cognitive Map (Oxford University Press).
5. Wills, T.J., Cacucci, F., Burgess, N., and O'Keefe, J. (2010). Development of the hippocampal cognitive map in preweanling rats. Science *328*, 1573–1576.
6. Langston, R.F., Ainge, J.A., Couey, J.J., Canto, C.B., Bjerknes, T.L., Witter, M.P., Moser, E.I., and Moser, M.B. (2010). Development of the spatial representation system in the rat. Science *328*, 1576–1580.
7. Tan, H.M., Bassett, J.P., O'Keefe, J., Cacucci, F., and Wills, T.J. (2015). The development of the head direction system before eye opening in the rat. Curr. Biol. *25*, 479–483.
8. Bjerknes, T.L., Langston, R.F., Kruge, I.U., Moser, E.I., and Moser, M.B. (2015). Coherence among head direction cells before eye opening in rat pups. Curr. Biol. *25*, 103–108.
9. Skaggs, W.E., Knierim, J.J., Kudrimoti, H., and McNaughton, B.L. (1995). A model of the neural basis of the rat's sense of direction. In Neural Information Processing Systems 7, S.J. Hanson, J.D. Cowan, and C.L. Giles, eds. (MIT Press), pp. 173–180.
10. Zhang, K. (1996). Representation of spatial orientation by the intrinsic dynamics of the head-direction cell ensemble: a theory. J. Neurosci. *16*, 2112–2126.
11. Redish, A.D., Elga, A.N., and Touretzky, D.S. (1996). A coupled attractor model of the rodent head direction system. Network *7*, 671.
12. Muessig, L., Hauser, J., Wills, T.J.T.J., and Cacucci, F. (2015). A Developmental Switch in Place Cell Accuracy Coincides with Grid Cell Maturation. Neuron *86*, 1167–1173.
13. Hardcastle, K., Ganguli, S., and Giocomo, L.M. (2015). Environmental boundaries as an error correction mechanism for grid cells. Neuron *86*, 827–839.
14. Taube, J.S., Muller, R.U., and Ranck, J.B., Jr. (1990). Head-direction cells recorded from the postsubiculum in freely moving rats. II. Effects of environmental manipulations. J. Neurosci. *10*, 436–447.
15. Yoganarasimha, D., Yu, X., and Knierim, J.J. (2006). Head direction cell representations maintain internal coherence during conflicting proximal and distal cue rotations: comparison with hippocampal place cells. J. Neurosci. *26*, 622–631.
16. Peyrache, A., Lacroix, M.M., Petersen, P.C., and Buzsáki, G. (2015). Internally organized mechanisms of the head direction sense. Nat. Neurosci. *18*, 569–575.
17. Tsanov, M., Chah, E., Noor, M.S., Egan, C., Reilly, R.B., Aggleton, J.P., Erichsen, J.T., Vann, S.D., and O'Mara, S.M. (2014). The irregular firing properties of thalamic head direction cells mediate turn-specific modulation of the directional tuning curve. J. Neurophysiol. *112*, 2316–2331.
18. Bassett, J.P., and Taube, J.S. (2001). Neural correlates for angular head velocity in the rat dorsal tegmental nucleus. J. Neurosci. *21*, 5740–5751.
19. Sharp, P.E., Tinkelman, A., and Cho, J. (2001). Angular velocity and head direction signals recorded from the dorsal tegmental nucleus of gudden in the rat: implications for path integration in the head direction cell circuit. Behav. Neurosci. *115*, 571–588.
20. Frohardt, R.J., Bassett, J.P., and Taube, J.S. (2006). Path integration and lesions within the head direction cell circuit: comparison between the roles of the anterodorsal thalamus and dorsal tegmental nucleus. Behav. Neurosci. *120*, 135–149.
21. Butler, W.N., Smith, K.S., van der Meer, M.A.A., and Taube, J.S. (2017). The Head-Direction Signal Plays a Functional Role as a Neural Compass during Navigation. Curr. Biol. *27*, 2406.
22. Hahnloser, R.H. (2003). Emergence of neural integration in the head-direction system by visual supervision. Neuroscience *120*, 877–891.
23. Stringer, S.M., Trappenberg, T.P., Rolls, E.T., and de Araujo, I.E.T. (2002). Self-organizing continuous attractor networks and path integration: one-dimensional models of head direction cells. Network *13*, 217–242.
24. Stratton, P., Wyeth, G., and Wiles, J. (2010). Calibration of the head direction network: a role for symmetric angular head velocity cells. J. Comput. Neurosci. *28*, 527–538.
25. McNaughton, B.L., Battaglia, F.P., Jensen, O., Moser, E.I., and Moser, M.B. (2006). Path integration and the neural basis of the 'cognitive map'. Nat. Rev. Neurosci. *7*, 663–678.
26. Fuhs, M.C., and Touretzky, D.S. (2006). A spin glass model of path integration in rat medial entorhinal cortex. J. Neurosci. *26*, 4266–4276.

27. Burak, Y., and Fiete, I.R. (2009). Accurate path integration in continuous attractor network models of grid cells. PLoS Comput. Biol. *5*, e1000291.
28. Widloski, J., and Fiete, I.R. (2014). A model of grid cell development through spatial exploration and spike time-dependent plasticity. Neuron *83*, 481–495.
29. Wills, T.J., Barry, C., and Cacucci, F. (2012). The abrupt development of adult-like grid cell firing in the medial entorhinal cortex. Front. Neural Circuits *6*, 21.
30. Seelig, J.D., and Jayaraman, V. (2015). Neural dynamics for landmark orientation and angular path integration. Nature *521*, 186–191.
31. Kim, S.S., Rouault, H., Druckmann, S., and Jayaraman, V. (2017). Ring attractor dynamics in the Drosophila central brain. Science *356*, 849–853.
32. Green, J., Adachi, A., Shah, K.K., Hirokawa, J.D., Magani, P.S., and Maimon, G. (2017). A neural circuit architecture for angular integration in Drosophila. Nature *546*, 101–106.
33. Turner-Evans, D., Wegener, S., Rouault, H., Franconville, R., Wolff, T., Seelig, J.D., Druckmann, S., and Jayaraman, V. (2017). Angular velocity integration in a fly heading circuit. eLife *6*, e23496.
34. Grieves, R.M., and Jeffery, K.J. (2017). The representation of space in the brain. Behav. Processes *135*, 113–131.
35. Alpeeva, E.V., and Makarenko, I.G. (2009). Perinatal development of the mammillothalamic tract and innervation of the anterior thalamic nuclei. Brain Res. *1248*, 1–13.
36. Alpeeva, E.V., and Makarenko, I.G. (2007). [Perinatal development of mammillotegmental connections in rats]. Ontogenez *38*, 86–93.
37. Bassett, J.P., Tullman, M.L., and Taube, J.S. (2007). Lesions of the tegmentomammillary circuit in the head direction system disrupt the head direction signal in the anterior thalamus. J. Neurosci. *27*, 7564–7577.
38. Muir, G.M., Brown, J.E., Carey, J.P., Hirvonen, T.P., Della Santina, C.C., Minor, L.B., and Taube, J.S. (2009). Disruption of the head direction cell signal after occlusion of the semicircular canals in the freely moving chinchilla. J. Neurosci. *29*, 14521–14533.
39. Lannou, J., Precht, W., and Cazin, L. (1979). The postnatal development of functional properties of central vestibular neurons in the rat. Brain Res. *175*, 219–232.
40. Grant, R.A., Mitchinson, B., and Prescott, T.J. (2012). The development of whisker control in rats in relation to locomotion. Dev. Psychobiol. *54*, 151–168.
41. Twyman, A.D., Newcombe, N.S., and Gould, T.J. (2013). Malleability in the development of spatial reorientation. Dev. Psychobiol. *55*, 243–255.
42. Hermer, L., and Spelke, E.S. (1994). A geometric process for spatial reorientation in young children. Nature *370*, 57–59.
43. Cheng, K., Huttenlocher, J., and Newcombe, N.S. (2013). 25 years of research on the use of geometry in spatial reorientation: a current theoretical perspective. Psychon. Bull. Rev. *20*, 1033–1054.
44. Harris, K.D., Henze, D.A., Csicsvari, J., Hirase, H., and Buzsáki, G. (2000). Accuracy of tetrode spike separation as determined by simultaneous intracellular and extracellular measurements. J. Neurophysiol. *84*, 401–414.
45. Bonnevie, T., Dunn, B., Fyhn, M., Hafting, T., Derdikman, D., Kubie, J.L., Roudi, Y., Moser, E.I., and Moser, M.B. (2013). Grid cells require excitatory drive from the hippocampus. Nat. Neurosci. *16*, 309–317.
46. Chen, G., Manson, D., Cacucci, F., and Wills, T.J. (2016). Absence of Visual Input Results in the Disruption of Grid Cell Firing in the Mouse. Curr. Biol. *26*, 2335–2342.
47. Zhang, K., Ginzburg, I., McNaughton, B.L., and Sejnowski, T.J. (1998). Interpreting neuronal population activity by reconstruction: unified framework with application to hippocampal place cells. J. Neurophysiol. *79*, 1017–1044.
48. Zar, J.H. (2010). Biostatistical Analysis, Fifth Edition (Prentice Hall).

STAR★METHODS

KEY RESOURCES TABLE

REAGENT or RESOURCE	SOURCE	IDENTIFIER
Chemicals, Peptides, and Recombinant Proteins		
Cresyl violet	Sigma Aldrich	C5042, http://www.sigmaaldrich.com/catalog/product/sigma/c5042?lang=en&region=US
Histoclear	National Diagnostics	HS-202, https://www.nationaldiagnostics.com/histology/product/histo-clear-ii
Thionin	Sigma Aldrich	88930, https://www.sigmaaldrich.com/catalog/product/sigma/88930
Experimental Models: Organisms/Strains		
Lister hooded rats	In house breeding (Charles River original source)	http://www.criver.com/products-services/basic-research/find-a-model/lister-hooded?loc=GB
Software and Algorithms		
Custom MATLAB routines	This paper	N/A
MATLAB	Mathworks. MA	RRID: SCR_001622, https://uk.mathworks.com/products/matlab.html
Other		
Single-screw microdrive	Custom made	N/A
Microwire (17um, platinum iridium)	California Fine Wire Company	Product code:100167, http://www.calfinewire.com/datasheets/100167-platinum10iridium.html
NanoZ plating equipment	Multichannel Systems	nanoZ, http://www.multichannelsystems.com/products/nanoz
Recording system (pre-amp and systems unit)	Axona	Product code: Dacq/USB64, http://axona.com/products
Omnetic connectors (microdrive assembly)	Genalog	Product code: A79026-001,http://genalog.com/genalog-linecard/omnetics/
2x16channel headstage preamplifiers	Axona	Product code: HS-116M1D, http://axona.com/products

CONTACT FOR REAGENT AND RESOURCE SHARING

Further information and requests for resources should be directed to and will be fulfilled by the Lead Contact, Francesca Cacucci (f.cacucci@ucl.ac.uk).

EXPERIMENTAL MODEL AND SUBJECT DETAILS

Subjects were 21 male Lister Hooded rats (RGD 2312466) aged P12-P21 weighing 18-29 g at the time of surgery. All procedures were approved by the UK Home Office, subject to the restrictions and provisions contained in the Animals Scientific Procedures Act of 1986. Litters were bred on site and implanted subjects remained with their mothers and litter-mates throughout the experimental period. Litters were housed in 42x32x21 cm cages furnished with nesting material and environmental enrichment objects, and maintained on a 12:12 hour light:dark schedule with lights off at 13:00. Litters were culled to 8 pups at P4 in order to minimize inter-litter variability. Implanted pups were separated from the litter for between 20 to 120 minutes per day for electrophysiological recordings. All procedures were conducted in accordance with the UK Animals Scientific Procedures Act (1986).

METHOD DETAILS

Surgery and electrodes

Rats were anaesthetised using 1%–3% isoflurane and buprenorphine via subcutaneous injection at 0.15mg/kg of body weight. Rats were implanted with 8 tetrodes consisting of HM-L coated 90% platinum/10% iridium 17 μm wire (California Fine Wire, Grover City, CA). The implanted apparatus weighed 1 g. Tetrode bundles were implanted into the ADN using the following stereotaxic coordinates: 1.7 mm posterior to bregma, 1.2mm lateral from the midline at bregma, and 4.2mm ventral from the skull surface at bregma.

Following surgery, rats were placed on a heating pad until they could move spontaneously and then were returned to the home cage. After experiments were completed, tetrode position was confirmed by transcardially perfusing the rat (4% formaldehyde in PBS) while the tetrodes remained in their final position, followed by brain sectioning at 30μm, and Nissl-staining of the resulting sections. See Figure S1A for example sections.

Single-unit recording

Following surgery, rats were allowed 24 hours recovery. Tetrode bundles were then advanced ventrally in increments of 62.5-250 μm/day. Experimental recording sessions began when any single unit neural activity could be identified. Single unit data was acquired using the DACQ system (Axona Ltd, St. Albans, UK). Position and directional heading was recorded using a 2-point tracking system consisting of 2 LEDs spaced 7 cm apart and attached to the headstage amplifier in a fixed orientation relative to the animals' head. Isolation of single units from tetrode-recorded data was performed manually on the basis of peak-to-trough amplitude or principal components, using the TINT software package (Axona Ltd., St Albans, UK) with the aid of KlustaKwik1 automated clustering [44].

Behavioral Testing

Single-unit recording trials took place in one of two square recording arenas. The 'standard box' had a side length of 62.5cm and was 50cm high, painted light gray, and placed on a black platform. The box was placed in the open laboratory, and distal visual cues were available in the form of the fittings and contents of the laboratory. The floor of the arena was not cleaned. There were no further polarizing cues placed within the recording arena. In order to ascertain whether cells which did not display a stable HD correlate in the standard box could be anchored to the laboratory frame in any other environment, we recorded the activity of the cells in a smaller square box ('small box': 20cm side length, 42cm high). The small box was placed on the same platform as the standard box and centered in the same location. In the majority of small box trials, the small box contained two polarizing cues, a 3D piece of wood (2cm x 4cm x 42cm), placed in the NW corner, and a sheet of polystyrene covering the E wall. Rats were subject to between 1 and 4 standard box trials (median 2), and 1 to 5 small box recording trials (median 2) per session (maximum total trials 7, median 3). As HD cells in the standard box were already mature by P15-16 (see also [5–7]), the oldest rats (P17-21) were not tested in the small box. Rat pups were kept in a separate holding box (40 × 40 × 25cm) furnished with bedding and a heating pad in between recording trials. A sub-set of rats (N = 14, contributing 351 HD cells) were also tested in a circular environment, from P14 onward. The circular environment was 79cm diameter, and (similarly to the standard box) was wooden, painted light gray, and placed on a black platform. A large plain white cue card (75cm x 1 m), illuminated by a 40W lamp was placed outside the environment, approximately 75cm away from its edge. No polarizing cues were placed inside the environment.

QUANTIFICATION AND STATISTICAL ANALYSIS

Construction of firing rate maps

To minimize artifactual correlates due to under-sampling of position, data was only included if the angular path length for the session exceeded the equivalent of 25 full head turns (values derived from the 5th percentile of the whole dataset). Positional (directional) data was sorted into 6° bins in the yaw plane. Following this, total dwell time, d, and spike count, s, for the whole trial was calculated for each directional bin. The binned position dwell time and spike count maps were then smoothed using a 30° boxcar filter, and the rate for each directional bin is defined as s/d.

Classification of single-units as HD cells

To minimize artifactual correlates due to under-sampling, only cells which fired at least 100 spikes in a recording session were included in further analysis. Single units were classified as HD cells if the mean resultant vector length (Rayleigh vector; RV) of the directional firing rate map exceeded a threshold defined as the 95th percentile of a population of RV scores derived from age- and brain area-matched spatially shuffled data [5]. Briefly, shuffled data was generated by shifting spike trains relative to position by a random amount between 20 s and trial duration minus 20 s, leaving the temporal structure of the spike train and the positional data otherwise unchanged. The shuffled data was then used to construct directional rate maps, as described above. This process was repeated a sufficient number of times for there to be 100,000 shuffled RV values for every 1-day age group, for each brain area. Single units with an RV $\geq$ 95th percentile of this shuffled population were defined as HD cells.

Quantitative analysis of directional signaling

'*% HD cells*' was defined as the number of neurons classified as HD cells divided by number of total cells recorded in an environment (standard or small box). The difference between proportions of HD cells in the standard and small boxes in each age bin was tested using a 2-sample Z-test for proportions. '*Intra-trial stability*' was defined as the correlation between spatially corresponding bins from the first and second half of a single trial, using only those bins in which firing rate > 0Hz in at least one half of the trial. The effects of age and environment on intra-trial stability and the RV of HD cells were tested initially by a 2-way ANOVA, followed by tests of simple main effects to assess differences between environments at particular ages.

Temporal and Spatial Relationships between Cells

Temporal cross-correlograms were defined as the cross-correlograms (constructed using the MATLAB 'xcorr' function) between time-binned histograms of the spike times of two neurons (bin width = 200ms). In order to standardize the height of the central peak (or depth of the central trough) and allow comparisons across all cell pairs, raw temporal cross-correlograms were normalized by the mean of a population of cross-correlograms (100 per pair) constructed with spike-shifted data. The 100 spike-shifted correlograms were constructed by shifting the spikes of one neuron along a set of linearly spaced intervals from 20sec to [trial duration - 20sec]. The mean normalized spike count of the central 1 s of the cross-correlogram was then used as a measure of the degree of offset (i.e., coupled or anti-coupled firing) between neurons. The degree of similarity between central 1 s spike counts in the large and small boxes was compared using linear regression. Only cell pairs in which both contributing cells were classified as HD cells in the small box, and neither were classified as HD cells in the standard box, were included in this analysis.

Time-windowed spatial cross-correlograms between pairs of neurons were constructed analogously to those in [45, 46] but with spatial firing being assessed with respect to head direction, rather than to 2-dimensional space. The spike times of one neuron (the 'reference' neuron) were used to define a series of 10 s time windows, each starting at the time of a reference neuron spike (head directions of 'reference neuron' were assigned a zero degrees value - windows were reduced in duration if a reference spike occurred within 10 s of the beginning or end of the trial). Within each window, a histogram was created of the head directions associated with the spiking of the second neuron (the 'test' neuron; histogram bin width = 6°), with directions being defined relative to the window center. The histograms of all windows were then summed, and smoothed (30° boxcar filter), producing an overall map of the directions in which the test neuron fired relative to the reference neuron, within a short time window. To control for uneven patterns of head rotation, the spike histogram was then divided by a summed, smoothed, histogram of all of the relative directional dwell times across all time windows: this defined the spatial cross-correlogram. To assess the degree of spatially coupled firing and the angle of directional offset, the spatial cross-correlogram (range -180° to 180° from the reference spike) was used to construct a polar rate map (analogous to that for normal head-directional firing), and the mean direction and mean resultant vector length (i.e., Rayleigh Vector) for this map were calculated. A large Rayleigh Vector indicates a strong spatial coupling over the time frame defined by the window. The degree of similarity between angular offsets in the standard and small boxes was compared using circular-circular regression. Only cell pairs for which both contributing cells were classified as HD cells in the small box, and neither were classified as HD cells in the standard box, were included in this analysis.

Bayesian Decoding

Decoding in the standard box was performed using standard Bayesian methods (see for example [12, 47]) barring the key difference that the function of a neuron's average firing with respect to direction ($f_i(\mathbf{x})$ in Equation 35, ref [7]), was derived from firing rate maps recorded in the small box trial. The final Bayesian estimate of position:

$$P(dir \mid spk) = P(spk \mid dir) * P(dir)$$

was therefore an estimate of the direction which current HD network activity was signaling, defined within the directional reference frame of the small box trial. See Figure S1 for a comparison of this approach with the standard method, decoding standard box position using $f_i(\mathbf{x})$ based on standard box rate maps. Only ensembles containing at least 9 HD cells in at least one small box trial were included (N = 6 ensembles). The decoding window was 1 s long, and non-overlapping windows were used. To estimate the angular velocity at which the decoded direction was moving, the decoded position was up-sampled to 50Hz, smoothed with a 5 s Gaussian kernel, and the angular velocity for each sample was estimated as the difference in direction at sample N and sample N+100 (i.e., across a 2 s time step). To produce a comparable estimate of actual head angular velocity (in order to correlate this with the decoded estimate), the actual head direction of the animal was averaged across 1 s long non-overlapping bins (mimicking the output of the decoding algorithm), then up-sampled and smoothed equivalently. The angular velocity was then estimated across 2 s time steps. For small box decoding $f_i(\mathbf{x})$ was based on rate maps from the small box trial. In this case, odd minutes of the trial were used to decode even minutes of observed spiking, and vice versa, such that at no point was spiking data used to decode itself. The relationship between actual and decoded angular velocity was tested using linear regression, and the difference between the slopes of these regression fits (between small and standard box) was tested using Student's t, analogously to testing for the differences between two means [48].

Estimation of coupled cell pairs in P12 data

To investigate the presence of temporally or spatially coupled neurons in P12 ensembles, we measured the proportions of temporal cross-correlograms with high or low central 1 s mean normalized correlation (indicating temporally coupled or anti-coupled firing, respectively) and spatial cross-correlograms with high RV scores (indicating consistent spatial firing offsets with the time-window). We defined 95% confidence limits for the chances of finding high or low coupling scores in non-HD cell data, by measuring the 5th and 95th percentiles of the distributions of scores in the population of all known nonHD-nonHD cell pairs that were recorded (N Pair = 3,634). This population was based on all pairs of recorded neurons that were not classified as HD cells, under experimental conditions when stable HD cells could be detected (small box at ages P13-14; standard box and small box at ages P15-P21. The over-representation of coupling scores beyond these limits in P12 data (significantly greater than 5%) was tested using a 1-sample Z-test for proportions.

Current Biology, Volume *28*

Supplemental Information

Self-Organized Attractor Dynamics
in the Developing Head Direction Circuit

Joshua P. Bassett, Thomas J. Wills, and Francesca Cacucci

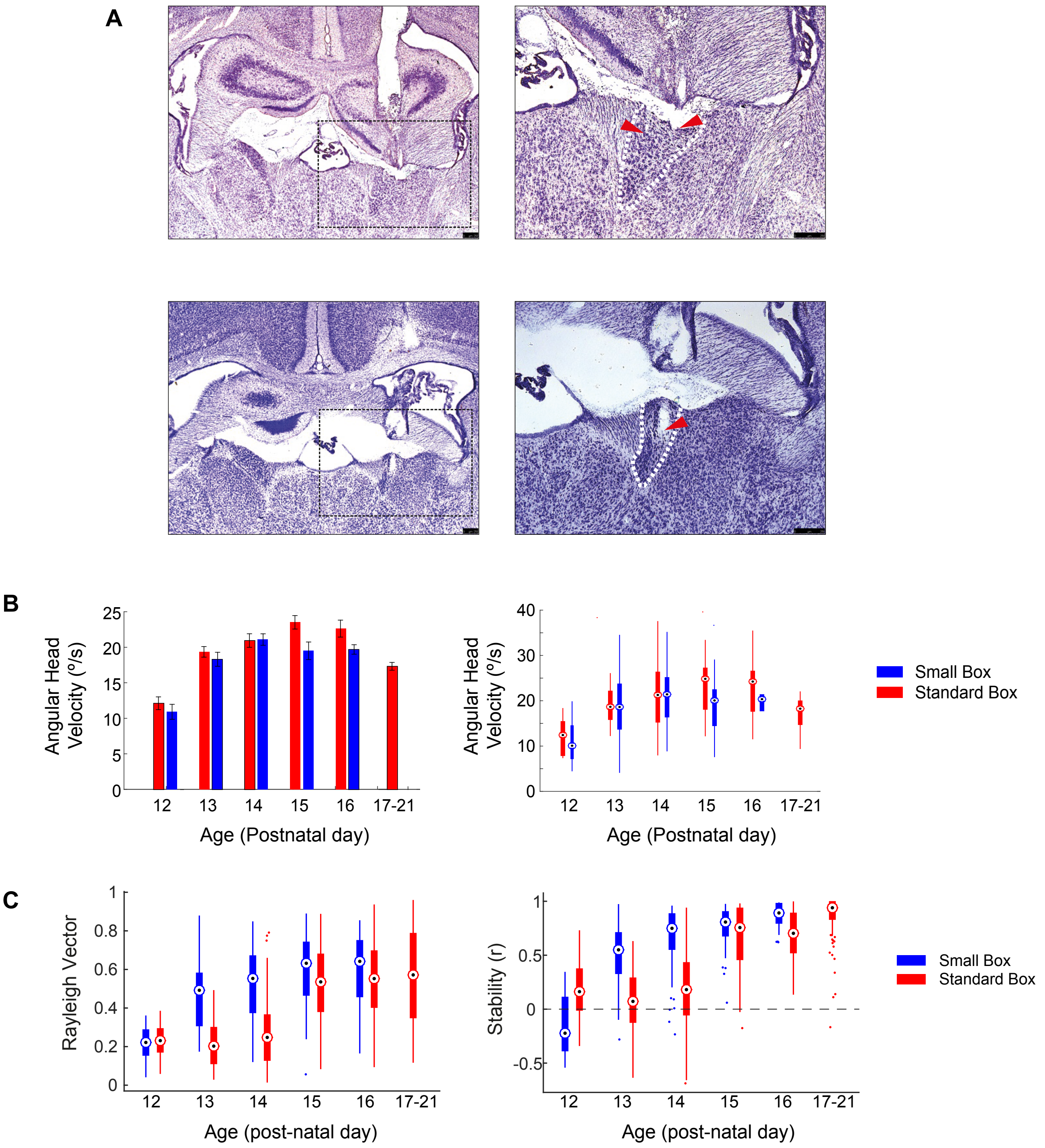

Figure S1. Anatomical recording locations, angular head velocity profiles and box-plots of head direction cell spatial firing properties, Related to Figure 1. (**A**) Nissl-stained brain sections (top, cresyl violet; bottom, thionin) showing representative recording locations in the Anterodorsal Nucleus of the thalamus (ADN). Boxes inset on right (black dashed lines) correspond to area of view on right. Red arrows mark the deepest location through which identifiable tetrode tracks passed. Approximate borders of ADN are outlined in white dashed lines in higher magnification pictures on right. Scale bars represent 250µm. (**B**) Absolute angular head velocity (AHV) does not differ significantly between the small and standard boxes, between P12 and P16. Left: bar chart shows the overall mean (±SEM) of the trial median AHVs in standard and small box, at each age. No significant differences between AHV in small and large box at any age can be detected (2-way ANOVA Age*Env; Env $F_{1,294}=2.86$, $p=0.09$, Age $F_{4,294}=21.8$, $p<0.0001$, Env*Age $F_{4,294}=1.47$, $p=0.21$). Right: the same data as panel B left, but displayed as box-and-whisker plots. For each group, black circle shows median, thick bar shows inter-quartile range (IQR) and thin bar shows the extent of the data, excluding outliers, which are shown as individual data points. Outliers were defined as lying further than 1.5*IQR beyond the nearest quartile. (**C**) Box-and-whisker plots showing distributions of Rayleigh Vector length and intra-trial stability for HD cells recorded in small and standard boxes P12 - P21 (same data as Figure 1B, middle, bottom). Format of box-and-whisker plots is as described in (B).

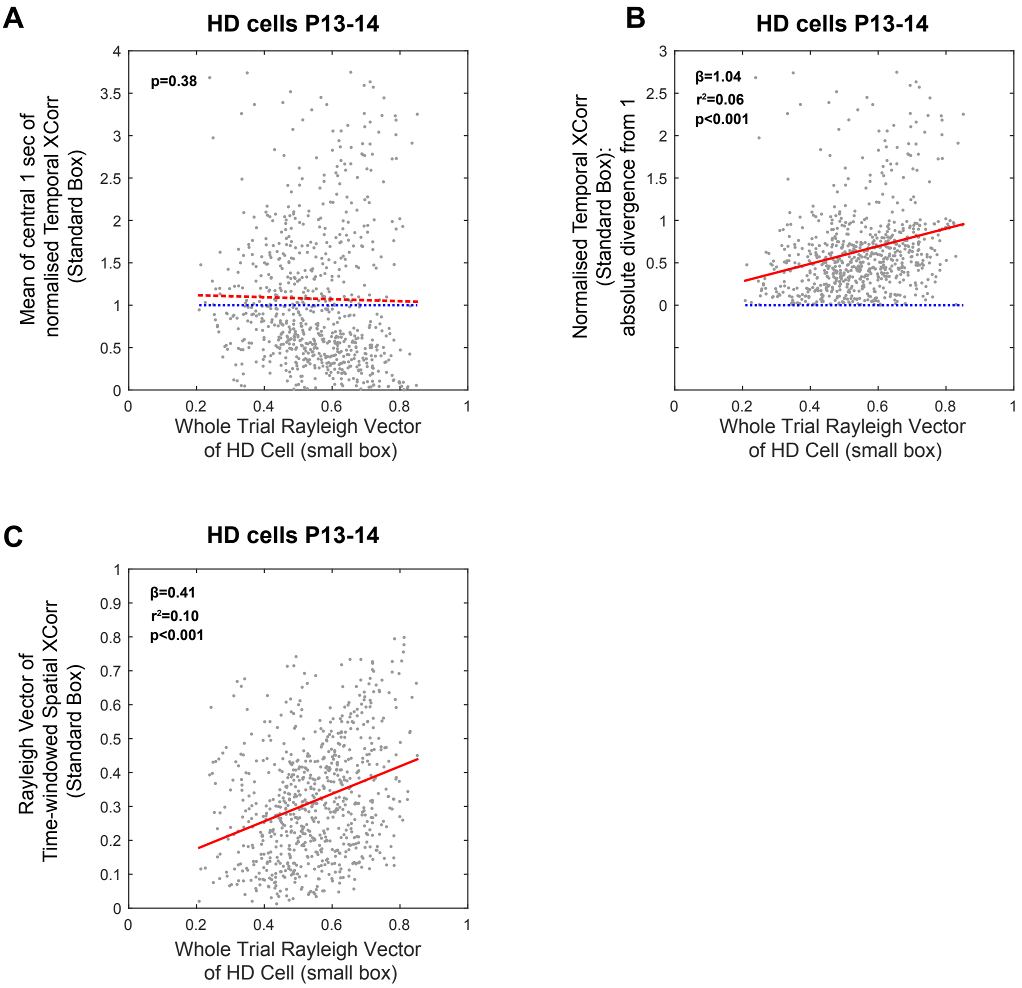

Figure S2. Relationship between head direction cell spatial tuning over a whole trial, and short time-scale temporal/spatial coupling scores, Related to Figure 3. Cross-correlogram scores indicating directionally coupled ADN cells in P12 rats (see Figure 3) cannot be explained by broader directional tuning of HD cells at younger ages. (**A**) Scatter plot showing the signed temporal coupling of known HD cell pairs, when drifting in the standard box at P13-14, versus the mean directional tuning of the cells over a whole trial, when stable in the small box (mean Rayleigh Vector length of directional rate maps; RV). There is no significant correlation between temporal coupling and RV ($p=0.38$, $\beta = -0.12$, $r^2=0.003$). (**B**) Temporal coupling scores diverge from 1 (indicating chance levels of coupling; blue dashed line) as whole trial RV increases, either increasing (indicating temporal co-firing) or decreasing (indicating temporal anti-coupling). We therefore also analysed the unsigned (absolute) temporal coupling of P13-14 HD pairs in standard box vs mean directional tuning of the cells in small box: there is a significant correlation between RV and divergence from a temporal coupling score of 1 (see Figure for stats), though the amount of variance explained by RV is low ($r^2 = 0.06$). (**C**) Scatter plot showing time-windowed spatial coupling of known HD cell pairs, drifting in the standard box at P13-14, versus the mean directional tuning of the cells over the whole trial in the small box (RV). Spatial coupling scores increase as mean RV increases (linear regression; see Figure for stats). Hence, the lower RV of HD cells seen in young rats cannot lead to artefactual over-estimation of temporal and spatial coupling scores at P12. We cannot rule out that the temporal and spatial cross-correlogram scores are under-estimating the numbers of directionally coupled cells at this age.

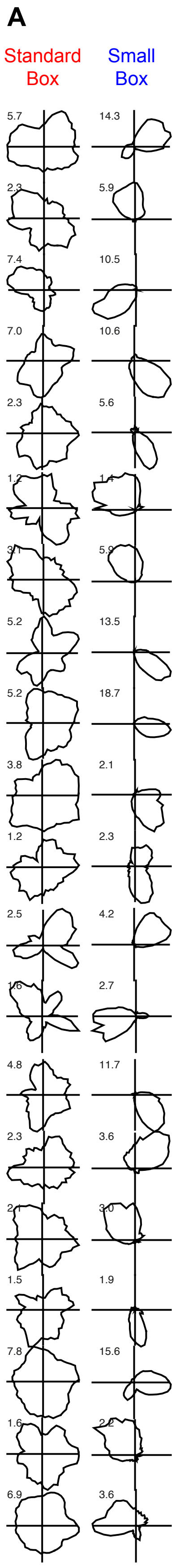

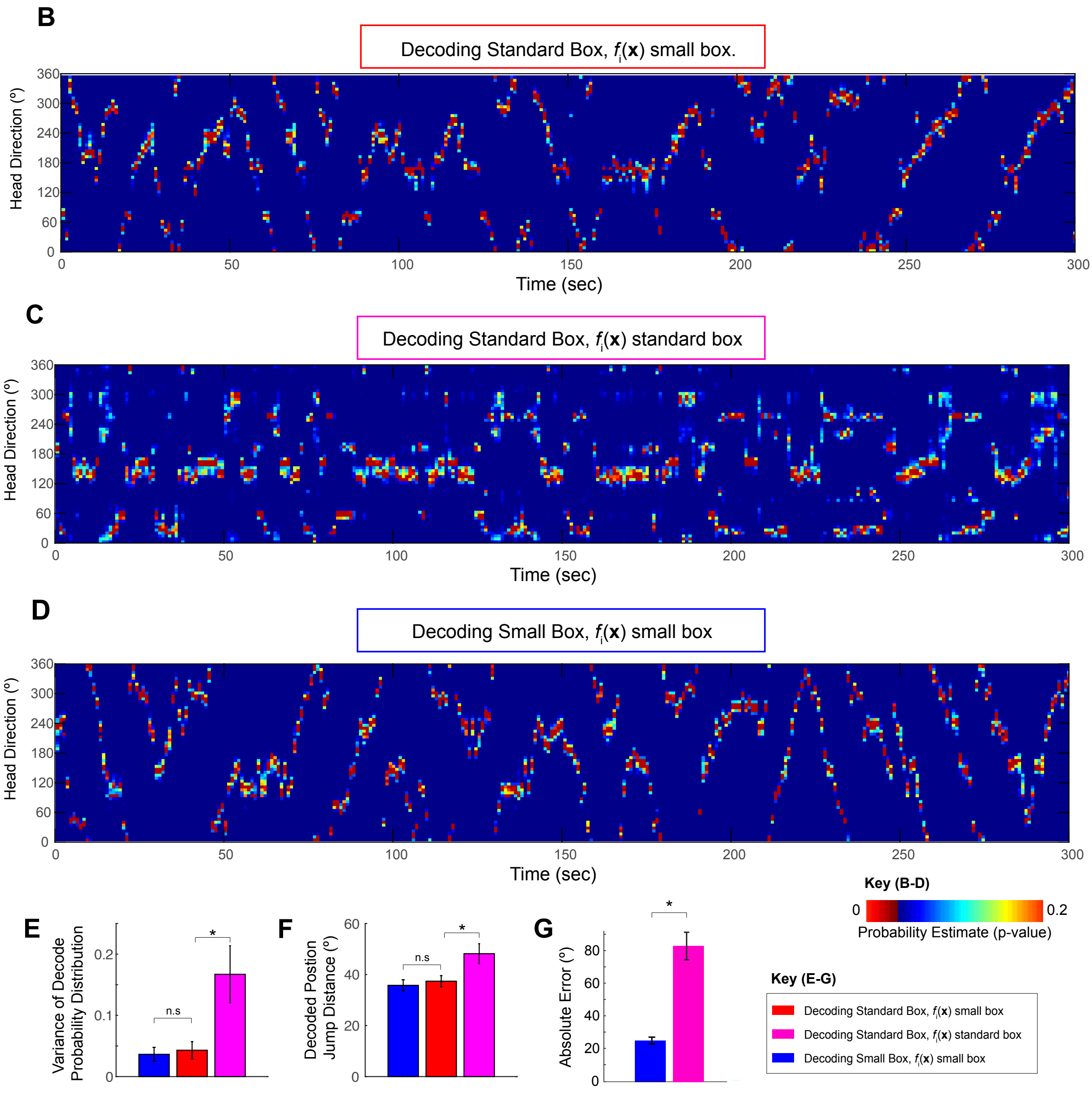

Figure S3. Validation of cross-trial (small/standard box) Bayesian decoding approach at P13-P14, Related to Figure 4. (**A**) Polar plots for all HD cells used for decoding in the example of Figure 2A, recorded in standard box and small box (P14). HD cells show stable directional signalling in the small box (right), but not in the standard box (left). (**B**) Full set of decoded probability estimates for example shown in Figure 2A. False colour image showing probability estimates of the rat facing in each direction (y-axis, bin size 6° width), computed from HD spiking in each decoding window (x-axis, window size = 1 sec). The function of average firing in a given direction for each cell ($f_i(x)$ where i represents the cell and x the direction) was based on polar plots derived from the small box trial. A coherent cluster of high probability is apparent in each decoding window and the estimated direction (maximum probability) moves smoothly between time bins, consistent with the presence of continuous attractor dynamics in the HD cell network, even though the HD network is not anchored to laboratory reference frame (see polar plots in (A) left column). (**C**) Full set of probability estimates obtained by decoding the same data as (A) and Figure 2A, in the case where $f_i(x)$ is based on the polar plots derived from the standard box trial. Consistent with the reduced spatial tuning and stability of the polar plots in the standard box, distributions of probability estimates within each decoding window display a high variance, and discontinuous jumps between temporally contiguous decoding windows are apparent. (**D**) 5 minute example of full probability estimates obtained decoding the small box trial (polar plots in (A), rightmost column). $f_i(x)$ was based on data derived from the same small box trial data (interleaved 60-sec time epochs were used for $f_i(x)$ construction and decoding, respectively, see methods for details). Comparison with (B) shows that the circular coherence of the probability estimates and the smoothness of the decoded trajectory are qualitatively similar whether decoding the small box, or the standard box using small box $f_i(x)$. (**E**) For all decoded ensembles (N=6), there is no significant difference in the circular coherence of probability within each window (measured by weighted circular variance), when comparing small box decoding and standard box decoding using small box $f_i(x)$. By contrast, standard box decoding using standard box $f_i(x)$ leads to significantly greater variance (ANOVA; $F_{2,15}$=6.51, p=0.009; Post-hoc HSD; small vs standard $f_i(x)$ small, p=0.98, standard $f_i(x)$ small vs $f_i(x)$ standard, p=0.021). (**F**) For all decoded ensembles, there is no significant difference in the smoothness of decoded trajectories (measured by the mean angular jump between consecutive decoded directions), when comparing small box decoding and standard box decoding using small box $f_i(x)$. By contrast, standard box decoding using standard box $f_i(x)$ leads to significantly greater average jumps (ANOVA; $F_{2,15}$=5.6, p=0.015; Post-hoc HSD; small vs standard $f_i(x)$ small, p=0.91, standard $f_i(x)$ small vs $f_i(x)$ standard, p=0.043). (**G**) Spatial accuracy of decoding, measured by absolute distance between actual and decoded head direction. Decoding the small box leads to absolute errors consistent with previously published studies[14]. By contrast, absolute error in the standard box is larger when using small box $f_i(x)$, indicating that the HD cells are not stably anchored to the testing environment reference frame (T-Test, t_5=6.1, p=0.002).

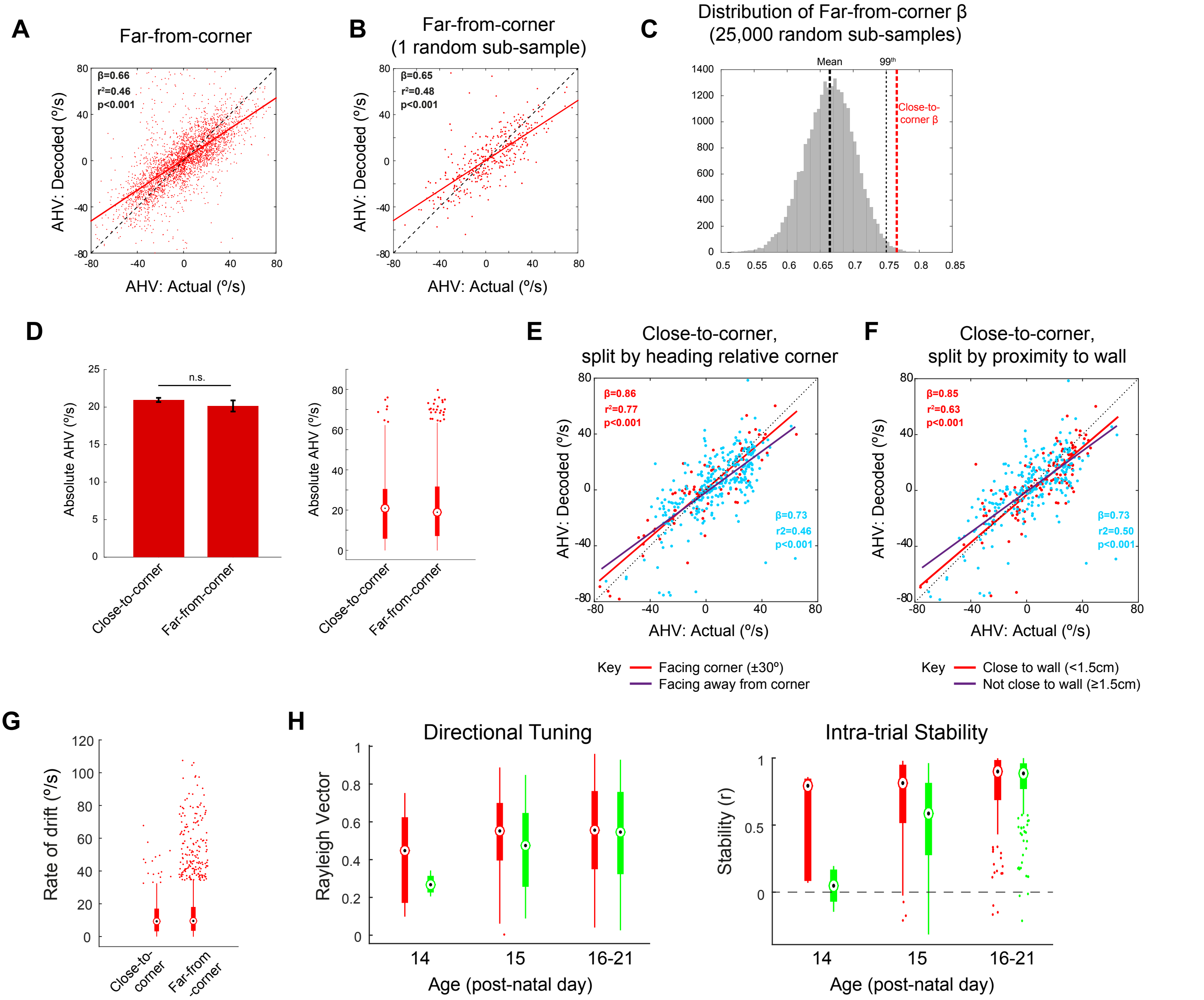

Figure S4. Control analyses for mitigation of angular head velocity (AHV) under-signalling close to corners, evidence for further mitigation of AHV under-signalling when facing corners, box-plots of rate of drift, and spatial properties of head direction cells in the circular environment, Related to Figure 4. (**A-C**) The difference between real versus decoded angular head velocity (AHV) regression slopes, for data recorded close-to-corners as compared to far-from-corners, is not due to different numbers of samples in the two groups. (**A**) Real versus decoded AHV regression for far-from-corner (same as Figure 4B, right). (**B**) The same data as (A), but randomly sub-sampled to have the same number of data points as the close-to-corner group (N=391). The regression slope is similar to the full dataset. (**C**) Distribution of regression slopes for 25,000 random sub-samples (to N=391) of the far-from-corner dataset. The mean regression slope of the sub-samples (0.667; heavy black dashed line) is very similar to that of the full dataset. The slope of the close-to-corner full dataset (0.774; red dashed line) lies beyond the 99th percentile of the sub-sampled slope distribution (light dashed black line). (**D**) Absolute AHV does not differ between close-to- and far-from-corner data. Right: bar chart shows mean (±SEM) AHV for each group. Means are not significantly different (t-test: $t_{(3478)}$=0.97, p=0.33). Left, box-and-whisker plot for the same data. For each group, black circle shows median, thick bar shows inter-quartile range (IQR) and thin bar shows the extent of the data, excluding outliers, which are shown as individual data points. Outliers were defined as lying further than 1.5*IQR beyond the nearest quartile. (**E-F**) AHV under-signalling is further reduced when rats are both close to corners, and (E) either facing the corner or (F) in very close proximity to the wall, suggesting that physical contact with the wall may help stabilise the HD signal. (**E**) Real versus decoded AHV linear regression for close-to-corner data, split according to whether the rat is facing the corner (looking directly at corner ±30°; red) or facing away from corner (sky blue). The facing corner regression slope is smaller than the non-facing slope, approaching significance at the p=0.05 level (t-test: $t_{(387)}$=1.593, p=0.054). The difference in slopes is unlikely due to the differences in sample size: when the facing away group data was randomly subsampled to match the facing group N (83), the actual slope for the facing corner group (β=0.862) was greater than the 90th percentile of the subsampled regression slopes (β=0.856). (**F**) Real versus decoded AHV linear regression for close-to-corner data, split according to whether the rat is in close proximity to a wall (when tracking lights on head are <1.5cm from wall; red) or further from a wall (sky blue). The close to wall regression slope is smaller than the further from wall slope, approaching significance at the p=0.05 level (t-test: $t_{(387)}$=1.631, p=0.051). The difference in slopes is unlikely due to the differences in sample size: when the facing away group data was randomly subsampled to match the facing group N (125), the actual slope for the facing corner group (β=0.854) is greater than the 90th percentile of the subsampled regression slopes (β=0.840). (**G**) Box-and-whisker plots for distributions of rate of drift, for data recorded close-to- and far-from-corners (same data as Figure 4C). Format of box-and-whisker plots is as described in (D). (**H**) Box-and-whisker plots for distributions of directional tuning (left) and intra-trial stability (right) of HD cells recorded in the standard box (red) and circle (green). (Same data as for Figure 4E). Format of box-and-whisker plots is as described in (D). There was an increase in the variability of the directional tuning of HD cells in the circle between P14 and P15 (F-test for RV: $F_{(4,98)}$=0.532, p=0.011), indicating that, at P14, only poorly tuned responses were observed, whereas by P15 both strong and poor directional tunings were observed. No other significant differences between group variances were observed (F-test, all p<0.1).
